# Learning Continuous Decomposable Models Using Mutual Information and Statistical Copulas

**DOI:** 10.3390/e28030293

**Published:** 2026-03-04

**Authors:** Luiz Desuó Neto, Henrique de Oliveira Caetano, Matheus de Souza Sant’Anna Fogliatto, Carlos Dias Maciel

**Affiliations:** 1 Department of Electrical Engineering, São Paulo State University (UNESP), Guaratinguetá 12516-410, SP, Brazil; carlos.maciel@unesp.br; 2 Department of Electrical and Computer Engineering, University of São Paulo (USP), São Carlos 13566-590, SP, Brazil; henriquecaetano1@usp.br (H.d.O.C.); matheusfogliatto@usp.br (M.d.S.S.F.)

**Keywords:** decomposable models, structure learning, mutual information, statistical copulas

## Abstract

Learning dependence graphs from multivariate continuous data is challenging when marginal distributions are heterogeneous, since likelihood-based nonparametric scores can be sensitive to smoothing choices and can confound marginal irregularities, including non-identifiability, with dependence. This work studies structure learning in the class of decomposable (chordal) Markov random fields, where junction tree factorizations enable tractable inference and local score updates. Our first contribution is a theoretical result showing that, under decomposability, mutual information can be expressed as a difference of clique/separator copula entropies, yielding a dependence-only decomposition aligned with the clique/separator structure. Building on this identity, we define an information-theoretic objective for decomposable graphs with a complexity penalty that preserves clique/separator additivity, and we derive closed-form local score differences for chordality-preserving single-edge insertions and deletions. To make the score computable from data, we instantiate clique/separator copula entropies using pseudo-observations and a probit-transformed kernel density estimator with predictive log score evaluation to mitigate boundary effects on the unit hypercube. The resulting nonparametric greedy procedure improves edge recovery accuracy on synthetic chordal benchmarks compared with a likelihood-driven nonparametric baseline, and it produces interpretable dependence summaries on an airway epithelial gene expression dataset. Concretely, this paper contributes (1) a decomposable mutual information identity via clique/separator copula entropies, (2) a copula information score with an additive complexity penalty for decomposable graphs, (3) a closed-form local score, enabling greedy chordal add or delete search, (4) a practical nonparametric copula entropy estimation pipeline, and (5) empirical gains on synthetic and real data.

## 1. Introduction

Dependence structure learning is increasingly used to convert high-dimensional continuous measurements into interpretable interaction networks that support downstream inference, hypothesis generation, and decision making [1]. In this context, both undirected MRFs and BNs are routinely used as structured representations of multivariate dependence, trading off interpretability, computational tractability, and (in the directed case) a natural language for causal hypotheses [2]. However, for continuous, heterogeneous data, one recurring difficulty is to combine a dependence score that is robust and marginally invariant with a graph class that supports scalable exact inference and local search updates without committing to a full directed learning pipeline [2]. In many scientific applications, this task must balance statistical fidelity with computational tractability because unconstrained structure learning is combinatorial, while the datasets motivating it are often large and heterogeneous [1].

Generally, calculating the partition function (normalizing factor that reestablishes the unit measure assumption from Kolmogorov’s probability axioms) of MRFs is an intractable problem [3]. Given the substantial computational burden, often exceeding feasibility, the cost-effectiveness of this strategy remains unclear. Without independent statistical interpretation for Gibbs potentials, attributing meaning to their parameters presents a significant challenge [4]. In contrast, the statistical meaning arises only from the joint probability density function, which is composed of their products. In light of the computational challenges and difficulty in interpreting the local parameters of Gibbs potentials, decomposable networks emerge as a relevant alternative due to their inherent advantages. For this reason, addressing non-chordal structure learning is intentionally left to work outside the scope of this work.

Although decomposable models admit both undirected and directed representations [4], this paper focuses on learning the undirected decomposable structure. This emphasis is deliberate; chordality yields a junction tree representation and clique/separator factorizations that enable scalable exact inference and genuinely local score updates during structure searching. BN learning (i.e., orienting edges and enforcing acyclicity, followed by a dedicated DAG search) can be treated as a complementary downstream step once a tractable, decomposable dependence scaffold has been learned. We do not develop that step here and instead refer to existing work on relating BNs and decomposable Markov networks [2,5].

A particularly attractive compromise is to restrict attention to decomposable (chordal) MRFs, since chordality yields a junction tree representation and enables clique/separator factorizations with efficient local computations [6]. This decomposable restriction has long served as a pragmatic foundation for scalable modeling, and it continues to motivate algorithmic research on both optimization and sampling over chordal graph spaces [6]. The work proposed by Olsson et al. [6] reinforced this perspective by developing explicit junction tree-based samplers, such as sequential sampling schemes that operate directly on junction tree structures to explore distributions over decomposable graphs. In parallel, recoverability analyses for tree-structured Markov random fields have clarified how noise and finite-sample effects can fundamentally limit structure identification even in low-treewidth settings [7].

However, these advances do not yet resolve the practical problem of structure recovery for continuous decomposable models under heterogeneous marginals. Junction tree samplers enable global exploration and uncertainty quantification over decomposable structures, but they do not by themselves provide a default, computationally dominant score for high-dimensional continuous data, where repeated clique/separator evaluations must be both stable and marginally robust [6]. At the same time, the sharpest modern identifiability and sample complexity results remain concentrated in tree-structured settings, leaving a gap in understanding how estimation error in local statistical primitives propagates through chordality-constrained search when the separator size exceeds one [7,8]. Finally, while the benchmarking infrastructure for structure learning is improving, end-to-end, decomposable graph recovery benchmarks for continuous regimes remain comparatively under-standardized, complicating reproducibility and fair comparison across score and search choices [1].

Despite recent progress in chordality-preserving search, learning continuous decomposable models remains fragile in practice because many widely used scores conflate dependence with one-dimensional marginal features and are sensitive to smoothing and boundary effects [9]. In particular, likelihood-driven nonparametric decomposable models based on clique-wise kernel density estimation avoid discretization but can become unstable under a bandwidth choice and can explain marginal irregularities by adding edges, which degrades structural interpretability [9].

Conversely, exact or near-exact combinatorial approaches for decomposable structure learning have been developed mainly for discrete formulations, where discretization or coarsening and state space growth can dominate the error budget when the underlying variables are genuinely continuous [10]. Many modern information-theoretic scoring ideas for continuous data have been developed primarily in the BN setting, where acyclicity and Markov equivalence shape both the score and the search space [2]. As a consequence, even when the target family is decomposable, it is nontrivial to obtain an undirected score that is simultaneously marginally invariant and junction tree-local, i.e., it admits clique/separator decomposition and inexpensive updates under chordality-preserving edits [9].

Moreover, on the BN side, widely used single-link look-ahead searches can fail in pseudo-independent domains, while multi-link look-ahead remedies are often prohibitively expensive because the DAG search space is far larger than the chordal and decomposable graph space, motivating intermediate-step strategies that first learn a decomposable MRF to guide BN learning at reduced complexity [2,5]. Together, these issues motivate a central open problem: to construct a dependence-oriented scoring criterion for continuous decomposable MRFs that is invariant to strictly monotone marginal reparameterizations, decomposes into clique/separator contributions to enable local score updates under chordality-preserving moves, and can be estimated robustly enough that repeated local comparisons do not translate smoothing artifacts into persistent false positives [2].

Copula theory offers a direct route to marginal invariance by separating marginal behavior from dependence through Sklar’s representation [11]. In particular, mutual information can be written as (negative) copula entropy, implying that a dependence score can be defined entirely with the copula density rather than the original marginal densities [12]. This identity has already been exploited in continuous structure learning for copula BNs via information-theoretic criteria and BIC-like penalties, suggesting that information-based scoring can be both practical and statistically targeted [13]. However, transferring this idea to decomposable undirected models requires a score that respects junction tree locality and admits efficient updates under chordality-preserving edge edits [9].

This paper addresses that gap by developing a copula information structure learning framework for continuous decomposable models that combines information theory, copula-based invariance, and chordal graph locality [12]. The proposed approach (1) rewrites the objective in terms of copula entropy contributions defined on clique and separator marginals, (2) introduces a CBIC-type complexity penalty that preserves clique/separator additivity, and (3) optimizes the resulting criterion via greedy chordality-preserving searching on decomposable graphs [13]. To support repeated evaluation of high-dimensional copula entropy terms, the method adopts a nonparametric copula density estimation strategy based on pseudo-observations and a probit transformation, which is designed to mitigate boundary bias on multidimensional unit hypercubes [14]. Algorithmically, chordality is maintained throughout the search using junction tree representations and dynamic chordal graph criteria so that each accepted move remains within the decomposable model class [15].

The significance of this contribution is twofold. It yields a dependence-focused learning criterion that is invariant to monotone marginal transformations, and it remains computationally aligned with decomposable graph search through local clique/separator updates [12]. Empirically, the resulting method is evaluated on controlled synthetic experiments (with a known chordal ground truth) and on a gene expression dataset, illustrating both improved structure recovery and practical applicability in noisy continuous measurements [1]. The approach taken in this work is to first establish a decomposable model identity that expresses mutual information through clique/separator copula entropies (Theorem 1) and then to use that identity to design a CBIC-type objective and a chordality-preserving greedy optimizer whose score differences depend only on the small set of affected clique/separator margins. We focus on learning the undirected decomposable structure; constructing an explicit BN from the learned chordal graph is complementary and is not pursued here (see [2,5]). The main contributions are as follows:

- Theoretical results: We prove a decomposable model identity showing that mutual information can be written as a difference of clique and separator copula entropies (Theorem 1), yielding an information decomposition aligned with a junction tree structure.
- New score design for decomposable graphs: Using Theorem 1, we define an information-theoretic objective for continuous decomposable models with a CBIC-type complexity penalty that preserves clique/separator additivity (Section 3.1), adapting the mutual information CBIC motivation from copula BNs [13] to the decomposable undirected settings.
- Local score delta for greedy chordal search: We derive a closed-form local update inspired by [9,15] for single-edge insertions and deletions under the proposed score (Section 3.1), adapting junction tree locality arguments from log-likelihood chordal and decomposable local search to the new copula information objective.
- Nonparametric estimation pipeline: For repeated evaluation of clique and separator terms, we instantiate the score using pseudo-observations and a probit-transformed, KDE-based copula entropy estimator with predictive log score evaluation (Section 3.3), leveraging the established copula entropy and boundary correction methodology [14,16].
- Empirical findings: On chordal synthetic benchmarks and a gene expression application, the resulting NG-CBIC procedure improves structure recovery (higher F1 and lower SHD) relative to likelihood-based nonparametric baselines under the same chordality-preserving search framework (Section 5).

The remainder of this paper is organized as follows. Section 2 develops the theoretical foundations, culminating in Theorem 1, which shows how mutual information can be expressed through clique/separator copula entropies in decomposable models. Section 3 turns this decomposition into a practical learning pipeline by defining the CBIC-type copula information score, deriving local score updates for chordality-preserving edge edits and specifying the nonparametric copula entropy estimator and greedy search procedure. Section 4 describes the experimental protocol and evaluation metrics, Section 5 reports the results on synthetic chordal benchmarks and a gene expression case study, and Section 6 concludes with limitations and directions for future work.

## 2. Theoretical Foundations

This section fixes the main probabilistic and graphical preliminaries used throughout the paper and clarifies how dependence is represented both analytically (via copulas) and structurally (via decomposable graphs). The guiding theme is that copulas separate marginal behavior from dependence, while decomposable (chordal) graphs make global dependence constraints tractable through clique/separator decompositions. These two perspectives will later be combined to produce information-theoretic measures that can be evaluated and updated locally during structure search.

### 2.1. Mutual Information as Statistical Copula Entropy

This subsection recalls the copula decomposition of a continuous multivariate distribution and uses it to rewrite mutual information in a form that depends only on the dependence structure. Under Sklar’s theorem, the joint law can be expressed through its marginals and a copula, isolating the part of the distribution that encodes rank dependence from the part that encodes one-dimensional behavior. This separation makes it natural to reinterpret information-theoretic quantities—originally defined via densities on $R^{d}$ or other continuous supports—in terms of the copula density on ${\left[0,1\right]}^{d}$.

Let (Ω,A,P) be a probability space, and let $X={\left(X_{1},\ldots ,X_{d}\right)}^{T}$ be a continuous random vector defined on this space with values in $R^{d}$. We denote its joint CDF by

$$F_{X}(x_{1},\ldots ,x_{d}):=P\left(X_{1}\leq x_{1},\ldots ,X_{d}\leq x_{d}\right),$$

which we abbreviate as F(x) for $x={\left(x_{1},\ldots ,x_{d}\right)}^{T}\in R^{d}$. Assume that *F* is absolutely continuous with respect to the Lebesgue measure $\lambda^{d}$ and that its joint density $p_{X}:R^{d}\to R_{+}$ satisfies

$$p_{X}\left(x\right)=\frac{\partial^{d}F\left(x\right)}{\partial x_{1}\cdots \partial x_{d}}.$$

For each component i∈{1,…,d}, we denote the marginal distribution function by $F_{i}\left(x_{i}\right):=P\left(X_{i}\leq x_{i}\right)$, with the corresponding marginal density $p_{i}\left(x_{i}\right)=F_{i}^{\prime }\left(x_{i}\right)$.

According to Sklar’s theorem [11], there exists a copula function $C:{\left[0,1\right]}^{d}\to \left[0,1\right]$ such that for all $x\in R^{d}$, we have

$$F_{X}\left(x_{1},\ldots ,x_{d}\right)=C\left(F_{1}\left(x_{1}\right),\ldots ,F_{d}\left(x_{d}\right)\right).$$

If the marginals $F_{i}$ are continuous, then the copula *C* is unique. Furthermore, if *C* is absolutely continuous with respect to the Lebesgue measure $\lambda^{d}$ on ${\left[0,1\right]}^{d}$, then it admits a copula density $c:{\left[0,1\right]}^{d}\to R_{+}$ defined by

$$c\left(u_{1},\ldots ,u_{d}\right)=\frac{\partial^{d}C\left(u_{1},\ldots ,u_{d}\right)}{\partial u_{1}\cdots \partial u_{d}}.$$

Under these assumptions, the joint density $p_{X}$ factorizes as follows:

(1) $$p_{X}\left(x_{1},\ldots ,x_{d}\right)=c\left(F_{1}\left(x_{1}\right),\ldots ,F_{d}\left(x_{d}\right)\right)\prod_{i=1}^{d}p_{i}\left(x_{i}\right).$$

The differential entropy of $X_{i}$ and of the joint vector X are

$$h\left(X_{i}\right)=-E[\log(p_{i}\left(X_{i}))\right],\;h\left(X\right)=-E[\log(p_{X}\left(X))\right].$$

Now, we define the distributional transform $U_{i}=F_{i}\left(X_{i}\right)$ for each i∈{1,…,d}. Each $U_{i}$ is uniformly distributed on [0,1], and the transformed random vector $U={\left(U_{1},\ldots ,U_{d}\right)}^{T}$ has a joint density $c\left(u\right)\equiv c\left(u_{1},\ldots ,u_{d}\right)$. The copula entropy is then defined by

$$h_{c}(U):=-E[\log(c(U)].$$

Finally, following Ma and Sun [12], if $h(X_{i})$ and h(X) are finite, then the mutual information of X can be expressed equivalently as follows:

(2) $$I\left(X\right)=\sum_{i=1}^{d}h\left(X_{i}\right)-h\left(X\right)=-\;h_{c}\left(U\right).$$

This identity demonstrates that the mutual information between continuous random variables equals the negative entropy of their associated copula density, thereby directly linking the dependence structure (via the copula) to information-theoretic principles. As a result, estimating mutual information reduces to estimating the (negative) entropy of the copula density. This perspective is particularly valuable for structure learning because it isolates conditional dependence patterns while remaining invariant to marginal artifacts.

### 2.2. Decomposable Models

This subsection reviews decomposable (chordal) undirected models as a structural restriction that enables exact clique/separator factorizations and efficient inference. The central point is that chordality is equivalent to the existence of a junction tree (clique tree) representation, in which global consistency is enforced through separator intersections (running intersection). This representation will later serve as the algorithmic scaffold for both scoring and admissible local graph edits.

PGMs describe how a joint distribution factorizes according to an underlying structure encoding conditional (in)dependencies. BNs are associated with DAGs, while MRFs use UGs as their representation. DMs occupy the intersection of these two classes; they are probability distributions that admit a perfect map in a chordal (triangulated) graph and hence can be represented both as DAGs and as undirected graphical models [4].

Let G=(V,E) be an undirected graph with a vertex set $V=\{v_{1},\ldots ,v_{d}\}$ and an edge set E⊆V×V, where each vertex $v_{i}\in V$ is associated with the component *i* of the random vector $X={\left(X_{1},\ldots ,X_{d}\right)}^{T}$. We will abbreviate sub-vectors using coordinate projections. For the *d*-dimensional random vector X and index set A⊂{1,…,d}, we define the projection $\pi_{A}:R^{d}\to R^{\left|A\right|}$ by $\pi_{A}\left(x\right):={\left(x_{i}\right)}_{i\in A}^{T}$ and write $X_{A}:=\pi_{A}\left(X\right)$ and $X_{-A}:=X_{A^{c}}$. The absence of an edge $\{v_{i},v_{j}\}\notin E$ corresponds to the pairwise Markov statement $X_{i}⫫X_{j}|X_{-\{i,j\}}$.

A subset K⊂V is called a clique if every pair of vertices in *K* is adjacent, and it is maximal if it is not strictly contained in any larger clique. Let $K\subset 2^{V}$ denote the collection of all maximal cliques of *G*. An undirected graph *G* is called chordal (or triangulated) if every cycle with a length of at least four has a chord, i.e., an edge joining two nonconsecutive vertices on the cycle. Moreover, *G* is decomposable if and only if it is chordal, and this is equivalent to the maximal cliques in the family K satisfying the running intersection property. Specifically, *G* is decomposable if and only if there exists an ordering $\left(K_{1},\ldots ,K_{m}\right)$ of the cliques in K such that for every l∈{2,…,m}, there exists an index η(l)<l (the predecessor of $K_{l}$) with

(3) $$S_{l}\equiv K_{l}\cap \overset{l-1}{\underset{k=1}{⋃}}K_{k}=K_{l}\cap K_{\eta (l)}.$$

In other words, the intersection of each clique $K_{l}$ with all previously listed cliques is contained in a single predecessor clique $K_{\eta (l)}$. The sets $S_{l}$ are called the separator sets, with the convention $S_{1}:=\emptyset$. For a decomposable graph *G*, such an ordering of maximal cliques exists and induces a junction tree representation of *G*.

Assume now that *G* is decomposable and encodes a Markov random field for X, with a joint density $p_{X}\left(x\right)>0$. With the ordering of maximal cliques $\left(K_{1},\ldots ,K_{m}\right)$ and separator sets $\left(S_{1},\ldots ,S_{m}\right)$ defined in Equation (3), the joint density factorizes with respect to the decomposable graph *G* if and only if

(4) $$p_{X}\left(x\right)=\prod_{l=1}^{m}p\left(x_{K_{l}\setminus S_{l}}|x_{S_{l}}\right)=\prod_{l=1}^{m}\frac{p(x_{K_{l}})}{p(x_{S_{l}})},$$

where $S_{1}=\emptyset$ so that the first factor reduces to $p(x_{K_{1}})$.

The factorization in Equation (4) shows how a strictly positive MRF on a decomposable graph can be written as a product of clique terms normalized by separator terms, avoiding double counting on overlaps. Importantly, this decomposition implies that many global quantities become sums (or differences of sums) over local subsets, provided they respect the same clique/separator structural constraints. The next subsection uses this idea to extend information measures defined on the copula into the decomposable setting.

### 2.3. Copula-Based Information Measures in Decomposable Models

This subsection combines the two previous threads; copulas encode dependence, and decomposable graphs organize multivariate objects through cliques and separators. The key technical step is to formalize how clique copulas induce separator copulas via marginalization (expressed here through γ marginals) so that the same junction tree overlaps are reflected at the copula level. Given this construction, information measures derived from copulas can inherit a clique/separator additivity principle.

Recalling the distributional transform $U_{i}:=F_{i}\left(X_{i}\right)$, we write $U={\left(U_{1},\ldots ,U_{d}\right)}^{T}$. Let *C* denote the copula of $X={\left(X_{1},\ldots ,X_{d}\right)}^{T}$, and let *c* denote its copula density. For any index set A⊂{1,…,d}, we write $U_{A}:=\pi_{A}\left(U\right)$ and denote by $C_{A}$ the distribution of $U_{A}$ with an (absolutely continuous) density $c_{A}$. To work with maximal clique and separator copulas in decomposable models, we now introduce the notion of a marginal copula.

**Definition** **1** (γ-marginal of a copula function [17])**.** *Let $C:{\left[0,1\right]}^{d}\to \left[0,1\right]$ be a d-dimensional copula function, and let $\gamma =\left\{j_{1},\ldots ,j_{r}\right\}\subset \left\{1,\ldots ,d\right\}$ with 1≤r≤d−1 and $j_{1}<\cdots <j_{r}$. The γ-marginal of C is the function $C^{\gamma }:{\left[0,1\right]}^{\left|A\right|}\to \left[0,1\right]$ defined by*

$$C^{\gamma }\left(u_{1},\ldots ,u_{r}\right):=C\left(v_{1},\ldots ,v_{d}\right),$$

*where it holds for each j∈{1,…,d} that*

$$v_{j}=\left\{\begin{array}{cc} u_{k}, & \mathrm{if}\;j=j_{k}\;\mathrm{for}\;\mathrm{some}\;k\in \left\{1,\ldots ,r,\right\}\\ 1, & \mathrm{otherwise}. \end{array}\right.$$

*If $C^{\gamma }$ is absolutely continuous, then we define its copula density by*

$$c^{\gamma }\left(u_{1},\ldots ,u_{r}\right):=\frac{\partial^{\left|A\right|}}{\partial u_{1}\cdots \partial u_{r}}\;C^{\gamma }\left(u_{1},\ldots ,u_{r}\right).$$

Now, let G=(V,E) be decomposable with maximal cliques $\left(K_{1},\ldots ,K_{m}\right)$ and separators $\left(S_{1},\ldots ,S_{m}\right)$ as shown in Equation (3), where $S_{1}=\emptyset$. For each clique $K_{l}\in K$, we fix a bijection $\alpha_{l}:\left\{1,\ldots ,|K_{l}|\right\}\to K_{l}$, which specifies the argument order used for the clique copula. For l≥2, we define the positional index set, selecting the separator coordinates inside clique $K_{l}$ via $\gamma_{l}:=\alpha_{l}^{-1}\left(S_{l}\right)\subset \left\{1,\ldots ,|K_{l}|\right\}.$ With this convention, the copula associated with the separator variables $U_{S_{l}}$ is the $\gamma_{l}$ marginal of the clique copula:

$$C_{S_{l}}\equiv C_{K_{l}}^{\gamma_{l}},\;\mathrm{and}\;\mathrm{hence}\;c_{S_{l}}\equiv c_{K_{l}}^{\gamma_{l}}.$$

**Proposition** **1** (Markov property under marginal transforms)**.** *Assume that each $F_{i}$ is continuous and strictly increasing, and define $U_{i}=F_{i}\left(X_{i}\right)$. Then, for any disjoint A,B,S⊆{1,…,d}, we have*

$$X_{A}⫫X_{B}|X_{S}\Leftrightarrow U_{A}⫫U_{B}|U_{S}.$$

*Consequently, if X is Markov with respect to G, then so is U.*

**Proof.**  Since $F_{i}$ is continuous and strictly increasing, it is a bijective Borel-measurable map R→R with a Borel-measurable inverse $F_{i}^{-1}$. Thus, $U_{i}=F_{i}\left(X_{i}\right)$ is $\sigma (X_{i})$-measurable and $X_{i}=F_{i}^{-1}\left(U_{i}\right)$ is $\sigma (U_{i})$-measurable, and hence

$$\sigma \left(U_{i}\right)\subseteq \sigma \left(X_{i}\right)\;\mathrm{and}\;\sigma \left(X_{i}\right)\subseteq \sigma \left(U_{i}\right),$$

such that $\sigma \left(X_{i}\right)=\sigma \left(U_{i}\right)$.

Now, we fix disjoint A,B,S⊆{1,…,d}. Because $X_{A}$ and $U_{A}$ generate the same σ field, similar to *B* and *S*, one has

$$\sigma \left(X_{A}\right)=\sigma \left(U_{A}\right),\;\sigma \left(X_{B}\right)=\sigma \left(U_{B}\right),\;\sigma \left(X_{S}\right)=\sigma \left(U_{S}\right).$$

Conditional independence of random vectors is defined in terms of their generated σ fields: $X_{A}⫫X_{B}|X_{S}\Leftrightarrow \sigma \left(X_{A}\right)⫫\sigma \left(X_{B}\right)|\sigma \left(X_{S}\right)$ [18].

Using the equalities of the σ fields above, this is equivalent to

$$\sigma \left(U_{A}\right)⫫\sigma \left(U_{B}\right)|\sigma \left(U_{S}\right),$$

which is to say that $U_{A}⫫U_{B}|U_{S}$.

The same argument in reverse shows the converse implication, and hence

$$X_{A}⫫X_{B}|X_{S}\Leftrightarrow U_{A}⫫U_{B}|U_{S}$$

for all disjoint A,B,S⊆{1,…,d}, completing the proof. □

Building upon this notion of γ marginals, the main structural message for copula-decomposable models is that the copula inherits the same clique/separator organization as the classical decomposable factorization in Equation (4). In a junction tree, adjacent cliques overlap only through their separator sets, so if one naively multiplies clique copula densities, the contribution of the shared variables is counted multiple times. The remedy is the familiar separator normalization; dividing by the corresponding separator copula density removes exactly the overcounted dependence associated with the overlap. Formally, this cancellation is justified by the running intersection property together with an induction along the chosen clique ordering. Variables that appear earlier can only reappear through separators and therefore vanish between the numerator and denominator terms, while the remainder sets $K_{l}\setminus S_{l}$ contribute only once. As a result, all marginal effects cancel out, leaving a factorization that isolates the dependence structure entirely through ratios of clique and separator copula densities. A complete, more standard proof (cast in the usual junction tree formalism) is provided in [19].

Equivalently, the same conclusion follows directly from the decomposable (junction tree) factorization applied to the copula-transformed variables. According to Proposition 1, the componentwise transform $U_{i}=F_{i}\left(X_{i}\right)$ preserves conditional independences, and thus U is Markov with respect to the same decomposable graph *G*. Any Markov distribution on a decomposable graph admits a junction tree marginal factorization into clique marginals divided by separator marginals (see Equation (4)). Since the joint density of U coincides with the copula density *c*, and for any *A* the marginal density of $U_{A}$ is $c_{A}$, applying this factorization to U yields the decomposable copula factorization in Equation (5). In particular, the global copula density admits the decomposable factorization

(5) $$c\left(u\right)=\prod_{l=1}^{m}\frac{c_{K_{l}}\left(u_{K_{l}}\right)}{c_{K_{l}}^{\gamma_{l}}\left(u_{S_{l}}\right)},\;u\in {\left[0,1\right]}^{d},$$

with the convention that the denominator equals 1 for l=1 (since $S_{1}=\emptyset$).

As an immediate consequence of Equation (5), mutual information admits a copula entropy representation that is compatible with the clique/separator structure of *G* [19].

**Theorem** **1** (Mutual information via copula entropy in decomposable models)**.** *Let $X={\left(X_{1},\ldots ,X_{d}\right)}^{T}$ be a random vector with continuous marginals, let $U={\left(U_{1},\ldots ,U_{d}\right)}^{T}$ with $U_{i}:=F_{i}\left(X_{i}\right)$, and let c denote the copula density of X. Assume that the dependence graph G is decomposable with maximal cliques $\left(K_{1},\ldots ,K_{m}\right)$ and separator sets $\left(S_{1},\ldots ,S_{m}\right)$ (with $S_{1}=\emptyset$) as shown in Equation *(3)* and that the copula density factorizes as shown in Equation *(5)*. For every non-empty A⊆{1,…,d}, let $c_{A}$ be the density of $U_{A}:=\pi_{A}\left(U\right)$ and define its copula entropy as*

$$h_{c_{A}}(U_{A}):=-E[\log(c_{A}\left(U_{A}))\right],$$

*whenever the expectation is finite.*

*If E[|log(c(U))|]<∞, $E[|\log(c_{K_{l}}\left(U_{K_{l}}\right)))|]<\infty$, and $E[|\log(c_{S_{l}}\left(U_{S_{l}}\right)))|]<\infty$ for all l∈{1,…,m}, then*

(6) $$I\left(X\right)=\sum_{l=1}^{m}h_{c_{S_{l}}}\left(U_{S_{l}}\right)-\sum_{l=1}^{m}h_{c_{K_{l}}}\left(U_{K_{l}}\right),$$

*with $c_{S_{l}}\equiv c_{K_{l}}^{\gamma_{l}}$.*

**Proof.**  Under the identity $I\left(X\right)=-h_{c}\left(U\right)$ from Equation (2), one has

I(X)=E[log(c(U))].

Using the decomposable copula factorization from Equation (5), we obtain

$$\log\left(c(U)\right)=\sum_{l=1}^{m}\log(c_{K_{l}}\left(U_{K_{l}})\right)-\sum_{l=1}^{m}\log(c_{S_{l}}\left(U_{S_{l}})\right),$$

where $c_{S_{l}}\equiv c_{K_{l}}^{\gamma_{l}}$ by construction and $S_{1}=\emptyset$ contributes no term.

Taking the expectations and using linearity (justified by the assumed integrability) yields

$$I\left(X\right)=\sum_{l=1}^{m}E[\log(c_{K_{l}}\left(U_{K_{l}}))\right]-\sum_{l=1}^{m}E[\log(c_{S_{l}}\left(U_{S_{l}}))\right].$$

Finally, since $E[\log(c_{A}\left(U_{A}\right))]=-h_{c_{A}}\left(U_{A}\right)$ by definition of copula entropy for each relevant margin, rearranging this gives Equation (6). □

The decomposable copula factorization in Equation (5) is the structural statement that makes the proposed learning algorithm possible; it rewrites a global dependence density as a product of clique copula terms divided by separator copula terms, matching the usual junction tree normalization logic. On this basis, mutual information decomposes into a difference of sums of copula entropies over separators and cliques, as stated in Theorem 1.

Taken together, the results in this section justify an information-centric representation of dependence that is simultaneously invariant to monotone marginal transforms and compatible with junction tree factorizations. This is precisely the combination needed for scalable learning; copula entropy links directly to mutual information, and decomposability converts global objects into sums of local clique and separator contributions. The remainder of the paper treats this decomposition not just as a theoretical identity but as a blueprint for defining scores and computing local updates under chordality-preserving moves. The next section leverages this compatibility to define a practical nonparametric scoring criterion and a greedy search strategy that maintains chordality at every step.

## 3. Nonparametric Structure Learning for Decomposable Models Using Information-Based Measures

This section turns the theoretical copula–graph compatibility into a concrete structure-learning procedure for decomposable graphs. The goal is to optimize a global objective that is motivated by mutual information but is computable from data using nonparametric copula entropy estimators. Because the search is restricted to chordal graphs, the algorithm can maintain a junction tree throughout and exploit clique/separator locality to evaluate score changes efficiently.

Let $X={\left(X_{1},\ldots ,X_{d}\right)}^{T}$ be a continuous random vector as defined previously. The objective is to learn a decomposable graph G=(V,E) that encodes the conditional independence structure of X. This is formulated as optimizing a global criterion derived from the copula-based information measures introduced in Theorem 1. The search is constrained to the space of chordal (decomposable) graphs and proceeds via local edge modifications that preserve decomposability.

### 3.1. Information-Theoretic Scoring Function

In this section, we define the population-level score that will drive structure learning, using copula entropy as the primitive building block. The definition is engineered to mirror the clique/separator decomposition of mutual information in decomposable models so that the score is both globally meaningful and locally decomposable. A CBIC-type penalty is then added to balance fit and complexity while preserving the same additivity pattern across cliques and separators.

Let *G* be a decomposable graph with maximal cliques $\left(K_{1},\ldots ,K_{m}\right)$ and separators $\left(S_{1},\ldots ,S_{m}\right)$ satisfying the running intersection property (Equation (3)), where $S_{1}=\emptyset$. For any nonempty index set A⊆{1,…,d}, recall the copula entropy $h_{c_{A}}\left(U_{A}\right)=-E[\log(c_{A}\left(U_{A}\right))]$. Define the functional

(7) $$\psi \left(A\right):=-h_{c_{A}}\left(U_{A}\right)=E[\log(c_{A}\left(U_{A}))\right].$$

In direct analogy to Equation (6), we define the global information of *G* as

(8) $$I\left(G\right):=\sum_{l=1}^{m}\psi \left(K_{l}\right)-\sum_{l=1}^{m}\psi \left(S_{l}\right).$$

According to Theorem 1, if the copula density *c* factorizes according to *G* as in Equation (5), then I(G)=I(X), representing the mutual information of the full vector.

Lasserre et al. [13] proposed an improved CBIC criterion for structure learning in copula BNs, in which the log-likelihood is rewritten in terms of mutual information (equivalently, copula entropy) contributions. Motivated by this construction, we introduce an analogous global information-based score for decomposable models. Assume that the graph complexity functional Λ(G) admits the clique/separator decomposition

(9) $$\Lambda \left(G\right):=\sum_{l=1}^{m}\lambda \left(K_{l}\right)-\sum_{l=1}^{m}\lambda \left(S_{l}\right),$$

for some nonnegative set functional $\lambda :2^{\left\{1,\ldots ,d\right\}}\to R_{+}$ (for instance, λ(A) may be the dimension of a parametric family or built from clique/separator dimensions λ(A)=|A|(|A|+1)/2).

Given the global information functional I(G) in Equation (8), we define the population CBIC-type score as

(10) $$S_{\mathrm{CBIC}}\left(G\right):=n\;I\left(G\right)-\frac{\log(n)}{2}\;\Lambda \left(G\right),$$

where *n* is the sample size. The scaling by *n* and log(n) mirrors the usual large-sample form of CBIC, while the leading term remains purely information-theoretic through I(G).

The resulting criterion $S_{\mathrm{CBIC}}\left(G\right)$ is designed such that (1) its leading term targets the dependence structure through information and (2) its penalty term can be chosen to reflect model capacity in a graph-local way [13]. This ensures that changing the graph only perturbs a small number of set function evaluations, which is crucial for greedy search. The next subsection makes this locality explicit by deriving closed-form score differences for single-edge updates.

### 3.2. Local Search and Score Decomposition

We incorporate the global score into a practical optimization procedure by focusing solely on single-edge modifications that preserve decomposability. The central observation is that under a junction tree representation, an admissible insertion or deletion only modifies a small neighborhood of cliques and separators [9], causing most score terms to cancel out in differences. As a result, it becomes possible to evaluate many candidate moves cheaply, without recomputing the entire score from scratch.

The search algorithm relies on evaluating the score change induced by a single edge addition or removal that preserves decomposability. Let *G* and $G^{\prime }$ be two decomposable graphs differing by exactly one such edge operation. Their respective score difference $S_{\mathrm{CBIC}}\left(G^{\prime }\right)-S_{\mathrm{CBIC}}\left(G\right)$ (Equation (10)) depends only on the cliques and separators whose composition changes such that most clique/separator contributions cancel out.

Let G=(V,E) be decomposable, and let $\left(K_{1},\ldots ,K_{m}\right)$ be a clique ordering satisfying the running intersection property with separators $S_{l}=K_{l}\cap K_{\eta (l)}$ as shown in Equation (3). Recall that the sum $\sum_{l=1}^{m}\psi \left(S_{l}\right)$ treats separators with their multiplicity induced by the chosen clique tree (equivalently, by the edges of a junction tree). This is relevant because different clique edges may yield identical separator sets as subsets of *V*.

A decomposable graph *G* may admit multiple junction tree (clique tree) representations and multiple running-intersection orderings. At the population level, when the copula density factorizes with respect to *G*, the resulting clique/separator decomposition (and hence I(G)) is a property of *G* and does not depend on the particular junction tree. In the finite-sample setting, however, $\hat{\psi }$ is an approximation, and numerical values can differ slightly across equivalent junction tree representations because separators enter the score with their edge-induced multiplicity. In our implementation, the score is therefore defined with respect to the current maintained junction tree T=(K,F); the separator contributions correspond to the multiset $\left\{K\cap K^{\prime }:\left(K,K^{\prime }\right)\in F\right\}$, and this same representation is used consistently throughout the search and for computing local score updates (see Section 3.4).

Denote by $G_{\mathrm{dec}}$ the class of decomposable graphs on *V*. For $G\in G_{\mathrm{dec}}$, we define its valid local neighborhood $N\left(G\right)\subset G_{\mathrm{dec}}$ as the set of decomposable graphs obtained from *G* by either inserting one edge $\{v_{i},v_{j}\}\notin E$ while preserving decomposability or deleting one edge $\{v_{i},v_{j}\}\in E$ while preserving decomposability. In practice, the admissibility of insert and delete moves can be checked using dynamic chordal graph criteria expressed on a maintained junction tree [15]. In particular, a deletion $\{v_{i},v_{j}\}\in E$ preserves decomposability if and only if $\left\{v_{i},v_{j}\right\}$ is contained in exactly one maximal clique of *G*, and an analogous junction tree criterion exists for insertion moves [9]. Assume that $G\in G_{\mathrm{dec}}$, and let $G^{\prime }\in N\left(G\right)$ be obtained by a single valid edge insertion. Assume that the move can be represented on some junction tree of *G* as follows. There exist two adjacent maximal cliques $K_{k}$ and $K_{l}$ with the separator

$$S=K_{k}\cap K_{l},$$

such that $v_{i}\in K_{k}\setminus S$ and $v_{j}\in K_{l}\setminus S$, and after inserting $\left\{v_{i},v_{j}\right\}$, a new maximal clique

$$K^{*}=S\cup \left\{v_{i},v_{j}\right\}$$

is created, replacing the separator *S* with the two separators $S\cup \{v_{i}\}$ and $S\cup \{v_{j}\}$ in the updated junction tree [9].

**Proposition** **2** (Local score update under edge insertion [9,15])**.** Let $G\in G_{\mathrm{dec}}$, and let $G^{\prime }$ be obtained from G by a single valid insertion of the edge $\{v_{i},v_{j}\}\notin E$, with the associated separator S and new clique $K^{*}:=S\cup \left\{v_{i},v_{j}\right\}$ as described above. Then, the score difference

$$\Delta_{ij}\left(G\right):=S_{\mathrm{CBIC}}\left(G^{\prime }\right)-S_{\mathrm{CBIC}}\left(G\right)$$

*can be written as*

(11) $$\Delta_{ij}\left(G\right)=\kappa \left(S\cup \{v_{i},v_{j}\}\right)-\kappa \left(S\cup \{v_{i}\}\right)-\kappa \left(S\cup \{v_{j}\}\right)+\kappa \left(S\right),$$

*where, for any nonempty A⊆{1,…,d}, we have*

(12) $$\kappa \left(A\right):=n\;\psi \left(A\right)-\frac{\log n}{2}\;\lambda \left(A\right),$$

*with ψ(A) as in Equation *(7)* and λ(A) being the local complexity term appearing in Equation *(9)*. In particular, $\Delta_{ij}\left(G\right)$ depends only on the four subsets S, $S\cup \{v_{i}\}$, $S\cup \{v_{j}\}$, and $S\cup \{v_{i},v_{j}\}$.*

**Proof.**  Write $S_{\mathrm{CBIC}}\left(G\right)$ in the clique/separator form in Equation (10), with separator multiplicities given by the chosen clique ordering (or, equivalently, by the junction tree). Under the described insertion move, all clique terms and all separator terms that are not incident to the modified junction tree edge cancel out in the difference $S_{\mathrm{CBIC}}\left(G^{\prime }\right)-S_{\mathrm{CBIC}}\left(G\right)$. The only changes are that the new clique $K^{*}$ is added; the old separator *S* is removed; and the two new separators $S\cup \{v_{i}\}$ and $S\cup \{v_{j}\}$ are added. Substituting these changes into Equation (10) yields Equation (11). □

Conversely, suppose that $\{v_{i},v_{j}\}\in E$ is contained in exactly one maximal clique *K* of *G* such that deleting $\left\{v_{i},v_{j}\right\}$ preserves decomposability, thus resulting in $G^{\prime }$. Let $S:=K\setminus \{v_{i},v_{j}\}$. Then, the deletion splits the clique *K* into two cliques, $S\cup \{v_{i}\}$ and $S\cup \{v_{j}\}$, connected by a separator *S*. In this case, the score change is the negative of Equation (11).

The update formula in Equation (11) provides a relevant operational result; it expresses $\Delta_{ij}\left(G\right)$ in terms of only four local subsets defined by the affected separator and the two incident vertices. Such local updates are what make greedy search over chordal graphs scalable when combined with efficient admissibility checks. The remaining task is to specify how the local information terms ψ(A) are estimated from data in a manner that is stable under the repeated evaluations required by the search procedure.

### 3.3. Nonparametric Copula Entropy Estimator

This section specifies the estimator used for the copula information functional, which is the statistical core of the scoring function. Because ψ(A) depends only on the copula of $X_{A}$, the procedure constructs pseudo-observations from empirical marginals, estimates a copula density on ${\left(0,1\right)}^{\left|A\right|}$ via transformation-based kernel smoothing, and evaluates a predictive log-score [12,14].

We adopt the nonparametric approach of Ma and Sun [12] and the related copula density-based method [14,16] to estimate the functional $\psi \left(A\right)=-h_{c_{A}}\left(U_{A}\right)$. Let $X^{\left(1\right)},\ldots ,X^{\left(n\right)}$ be an IID sample of $X={\left(X_{1},\ldots ,X_{d}\right)}^{T}$, and fix a nonempty index set A⊆{1,…,d}. Since ψ(A) depends only on the copula of $X_{A}$, the estimation reduces to constructing pseudo-observations for $U_{A}$ and estimating the corresponding copula density and copula entropy [12,20].

We partition the training index set $J_{\mathrm{tr}}$ into *k* disjoint folds $J_{1},\ldots ,J_{k}$ and define, for each fold r∈{1,…,k}, the corresponding CV training index set:

$$J_{-r}:=J_{\mathrm{tr}}\setminus J_{r}.$$

We then define the indicator function of a subset B⊆D as $\chi_{B}:D\to \left\{0,1\right\}$ with

$$\chi_{B}\left(b\right):=\{\begin{array}{cc} 1, & \mathrm{if}\;b\in B,\\ 0, & \mathrm{otherwise}. \end{array}$$

For each i∈{1,…,d} and each fold r∈{1,…,k}, we define the empirical marginal CDF

$${\hat{F}}_{i,r}\left(x\right):=\frac{1}{|J_{-r}|}\sum_{s\in J_{-r}}\chi_{\left(-\infty ,x\right]}\left(X_{i}^{\left(s\right)}\right),\;x\in R.$$

To obtain pseudo-observations in the unit interval that remain strictly away from the endpoints and are therefore suitable for probit transformation, we follow the standard pseudo-observation scaling used in copula estimation and add an explicit truncation. For each observation index t∈{1,…,n}, we define the fold *r* pseudo-observations by

$${\hat{U}}_{i,r}^{\left(t\right)}:=\min\;\left\{1-\varepsilon ,\;\max\;\left\{\varepsilon ,\;\frac{|J_{-r}|}{|J_{-r}|+1}\;{\hat{F}}_{i,r}\;\left(X_{i}^{\left(t\right)}\right)\right\}\right\}.$$

The factor $|J_{-r}\left|/(\right|J_{-r}\left|+1\right)$ is equivalent to the pseudo-observation construction based on marginal ranks, which is widely used in semiparametric copula inference [14]. The additional truncation at ε>0 is a deterministic numerical convention that ensures ${\hat{U}}_{i,r}^{\left(t\right)}\in \left[\varepsilon ,1-\varepsilon \right]$. This guarantees that the maximum observation in a fold never maps exactly to one and, similarly, that the minimum never maps exactly to zero.

The truncation is essential because the probit transformation $\Phi^{-1}$ is only defined on the open interval (0,1), with limiting behavior

$$\lim_{u\to 0^{+}}\Phi^{-1}\left(u\right)=-\infty ,\;\lim_{u\to 1^{-}}\Phi^{-1}\left(u\right)=+\infty .$$

Applying the probit transform to pseudo-observations exactly at zero or one would therefore yield infinite values, rendering kernel density estimation impossible. By enforcing ${\hat{U}}_{i,r}^{\left(t\right)}\in \left[\varepsilon ,1-\varepsilon \right]$, the transformed observations ${\hat{Z}}_{i,r}^{\left(t\right)}=\Phi^{-1}\left({\hat{U}}_{i,r}^{\left(t\right)}\right)$ are guaranteed to be finite real numbers for all sample points, ensuring the probit transformation remains numerically well-defined throughout the procedure [14]. We define the corresponding vectors and subvectors by

$${\hat{U}}_{r}^{\left(t\right)}:=({{\hat{U}}_{1,r}^{\left(t\right)},\ldots ,{\hat{U}}_{d,r}^{\left(t\right)})}^{T},\;{\hat{U}}_{A,r}^{\left(t\right)}:=\pi_{A}({\hat{U}}_{r}^{\left(t\right)})\in {\left(0,1\right)}^{\left|A\right|}.$$

Kernel smoothing on ${\left(0,1\right)}^{\left|A\right|}$ is prone to boundary bias. Following Geenens et al. [14], we therefore apply the probit transform component-wise. Let Φ and ϕ denote the standard normal CDF and PDF, respectively, and define

$${\hat{Z}}_{i,r}^{\left(t\right)}:=\Phi^{-1}\;({\hat{U}}_{i,r}^{\left(t\right)}),\;{\hat{Z}}_{r}^{\left(t\right)}:=({{\hat{Z}}_{1,r}^{\left(t\right)},\ldots ,{\hat{Z}}_{d,r}^{\left(t\right)})}^{T}\in R^{d},\;{\hat{Z}}_{A,r}^{\left(t\right)}:=\pi_{A}({\hat{Z}}_{r}^{\left(t\right)})\in R^{\left|A\right|}.$$

Let $\theta =\left(\theta_{1},\ldots ,\theta_{d}\right)\in R_{+}^{d}$ be a global diagonal bandwidth vector, and write $\theta_{A}:=\pi_{A}\left(\theta \right)$. We define the Gaussian kernel on $R^{\left|A\right|}$ by

$$g\left(z|\theta_{A}\right)={\left(2\pi \right)}^{-|A|/2}{\left|\mathrm{diag}(\theta_{A})\right|}^{-1/2}\exp(-\frac{1}{2}z^{T}\;\mathrm{diag}{\left(\theta_{A}\right)}^{-1}\;z).$$

For each fold *r* and subset A⊆{1,…,d}, define the probit transformation KDE estimator of the copula density $c_{A}$ by

(13) $${\hat{c}}_{A,r}\left(u_{A}|\theta_{A}\right):=\frac{\frac{1}{|J_{-r}|}\sum_{s\in J_{-r}}g\;(z_{A}-{\hat{Z}}_{A,r}^{\left(s\right)}|\theta_{A})}{\prod_{i\in A}\phi \left(z_{i}\right)},\;u_{A}\in {\left(0,1\right)}^{\left|A\right|},$$

where $z_{i}=\Phi^{-1}\left(u_{i}\right)$ for i∈{1,…,d}, $z={\left(z_{1},\ldots ,z_{d}\right)}^{T}\in R^{d}$ and the corresponding sub-vector on margin index set *A* is $z_{A}=\pi_{A}\left(z\right)$. The denominator in Equation (13) is the Jacobian factor arising from the change in variables between the probit-transformed space and the copula domain. We then define the estimator of ψ(A) as the average predictive log-score per observation:

(14) $${\hat{\psi }}_{\mathrm{KDE}}\left(A\right):=\frac{1}{n}\sum_{r=1}^{k}\sum_{t\in J_{r}}\log{\hat{c}}_{A,r}\;({\hat{U}}_{A,r}^{\left(t\right)}|\theta_{A}).$$

For each fold *r*, the empirical marginals ${\hat{F}}_{i,r}$ and the kernel centers ${\hat{Z}}_{A,r}^{\left(s\right)}$ are estimated using the training indices $J_{-r}$ only, while the predictive log-score averages $\log{\hat{c}}_{A,r}\left({\hat{U}}_{A,r}^{\left(t\right)}|\theta_{A}\right)$ over the held-out indices $t\in J_{r}$. This construction ensures that the predictive log-score is strictly out of fold and avoids information leakage from test folds into the fitted copula model.

Following the predictive-assessment KDE approach of Schwaighofer et al. [9], we select a single global bandwidth vector θ before the structure search and subsequently keep it fixed. Here, $\theta =\left(\theta_{1},\ldots ,\theta_{d}\right)\in R_{+}^{d}$ is a vector with one component per dimension, and for any subset *A*, we use the restriction $\theta_{A}=\pi_{A}\left(\theta \right)$ in Equation (13). The same global vector is shared across all cliques and separators.

We choose θ by maximizing a leave-one-out CV predictive log-score on the probit-transformed full training sample ${\left\{{\hat{Z}}^{\left(t\right)}\right\}}_{t\in J_{\mathrm{tr}}}\subset R^{d}$ without conditioning on a specific subset *A*. For numerical convenience, we optimize over the log-bandwidth parameters $\xi \in R^{d}$, defined component-wise by $\xi_{j}:=\log\left(\theta_{j}\right)$, so that θ is automatically in $R_{+}^{d}$. We solve a box-constrained problem using an L-BFGS-B quasi-Newton method [21,22,23]:

$$\hat{\xi }\in \underset{\xi \in [\ell ,h]}{arg max}\sum_{t\in J_{\mathrm{tr}}}\log\;\left(\frac{1}{|J_{\mathrm{tr}}|-1}\sum_{\begin{array}{c} s\in J_{\mathrm{tr}}\setminus \{t\} \end{array}}g\;({\hat{Z}}^{\left(t\right)}-{\hat{Z}}^{\left(s\right)}|\exp\left(\xi \right))\right),\;\hat{\theta }=\exp\left(\hat{\xi }\right).$$

The box constraints are chosen component-wise as

$$\ell_{j}=\log\;\left(\hat{\mathrm{Var}}\left(Z_{j}\right)\;\beta_{\min}\right),\;h_{j}=\log\;\left(\hat{\mathrm{Var}}\left(Z_{j}\right)\;\beta_{\max}\right),$$

for fixed constants $0<\beta_{\min}<\beta_{\max}$, where $\hat{\mathrm{Var}}\left(Z_{j}\right)$ is the empirical variance of the *j*th coordinate in ${\left\{{\hat{Z}}^{\left(t\right)}\right\}}_{t\in J_{\mathrm{tr}}}$. The optimizer is terminated when either a prescribed maximum number of iterations is reached or the relative change in the objective falls below a tolerance parameter.

This leave-one-out CV optimization is performed once before structure learning. In all experiments reported in Section 4, its wall clock time was negligible compared with the subsequent greedy search, which requires repeated evaluation of ${\hat{\psi }}_{\mathrm{KDE}}\left(A\right)$ for many overlapping subsets *A*.

The truncation ${\hat{U}}_{i,r}^{\left(t\right)}\in \left[\varepsilon ,1-\varepsilon \right]$ guarantees that the probit transform ${\hat{Z}}_{i,r}^{\left(t\right)}=\Phi^{-1}\left({\hat{U}}_{i,r}^{\left(t\right)}\right)$ is always finite and avoids singularities in the Jacobian factor $\prod_{i\in A}\phi {\left(z_{i}\right)}^{-1}$. In the computation of the KDE sums entering the leave-one-out CV objective and ${\hat{c}}_{A,r}$, we evaluate log-densities using log-sum-exp identities to avoid numerical underflow in the tails and for large values of |A|.

To further guard against numerical artifacts when ${\hat{c}}_{A,r}$ is extremely small, we apply a fixed-density floor. Throughout, we replace

$$\log{\hat{c}}_{A,r}\left(u_{A}|\theta_{A}\right)\;\leftarrow \;\max\;\left\{\log{\hat{c}}_{A,r}\left(u_{A}|\theta_{A}\right),\;\log\delta_{\mathrm{floor}}\right\}$$

for a small constant $\delta_{\mathrm{floor}}>0$. If a candidate evaluation yields non-finite values due to numerical issues, then we treat that candidate as inadmissible and deterministically prevent that configuration from being selected during greedy search. Because $\Delta_{ij}$ in Equation (11) depends only on differences in a small number of local terms, and the same flooring rule is applied uniformly across all candidate edges and graphs, this safeguard only affects terms that are already well below machine precision and did not lead to unstable edge decisions in our empirical study.

The estimator ${\hat{\psi }}_{\mathrm{KDE}}\left(A\right)$ inherits the standard large-sample properties of probit-transformed kernel copula density estimators. Under suitable smoothness conditions on $c_{A}$, and for bandwidths θ shrinking at appropriate rates, ${\hat{c}}_{A,r}$ is pointwise consistent (and, on compact subsets of the interior, uniformly consistent), with asymptotic bias and variance governed by the usual kernel-smoothing orders in the transformed domain [14,24]. Moreover, consistency of the plug-in predictive log-score for the copula information functional $\psi \left(A\right)=E[\log c_{A}\left(U_{A}\right)]$ holds under additional regularity, ensuring that the logarithm is well behaved. For example, $c_{A}$ is strictly positive on the interior and $E|\log c_{A}\left(U_{A}\right)|<\infty$, together with sufficiently strong (e.g., uniform-on-compacts) convergence of ${\hat{c}}_{A,r}$ so that $\log{\hat{c}}_{A,r}\to \log c_{A}$ on those regions [25,26].

The use of *k*-fold out-of-fold predictive log-scores rather than exact leave-one-out optimization modifies the fitted estimators through the smaller training size in each fold and hence can affect finite-sample variability (and, slightly, the finite sample bias), but for fixed *k* values, the difference is asymptotically negligible as n→∞ in standard cross-validatory logarithmic assessment settings [27,28]. The empirical marginal pseudo-observation construction implies that ${\hat{\psi }}_{\mathrm{KDE}}\left(A\right)$ is invariant under strictly monotone transformations of each marginal $X_{i}$, and thus the resulting score depends only on the copula of $X_{A}$ [14]. Finally, the endpoint truncation ${\hat{U}}_{i,r}^{\left(t\right)}\in \left[\varepsilon ,1-\varepsilon \right]$ ensures that the probit transform and its Jacobian factor remain finite; if ε→0, and $\log c_{A}\left(U_{A}\right)$ is integrable, then the truncation perturbs the target functional by a vanishing amount as $|J_{-r}|\to \infty$, akin to the trimming and truncation devices commonly used to control log-density singularities in kernel-based entropy estimation [25,26].

### 3.4. Search over Decomposable Structures

This subsection details the optimization strategy: a greedy forward and backward procedure over chordal graphs that enforces decomposability at every step. The junction tree is treated as the central state representation, since it simultaneously encodes the clique/separator structure for scoring and supports graph-theoretic queries for deciding whether an insertion or deletion is admissible. The algorithm is therefore best viewed as an interplay between local information gains (via $\Delta_{ij}\left(G\right)$) and local structural feasibility (via dynamic chordal criteria).

We optimize the global score I(G) in Equation (10) over $G_{\mathrm{dec}}$ via the greedy stepwise algorithm of Schwaighofer et al. [9], which alternates forward selection and backward elimination within a single run while preserving decomposability (chordality) at every step. In the original work [9], the score is a predictive assessment criterion based on a cross-validated log-likelihood for a decomposable kernel density estimator, where the clique/separator factorization enables local accumulation; however, the search mechanism is agnostic to the particular scoring functional. Starting from the empty graph, each iteration evaluates admissible single-edge insertions and deletions and applies the move that yields the largest score improvement, with the central design requirements being that valid local moves can be enumerated efficiently within $G_{\mathrm{dec}}$ and that each move admits a local score update as shown in Equation (11). Because unrestricted edge edits may violate chordality, candidate moves are explicitly restricted to chordality-preserving operations using a scheme inspired by fully dynamic chordal graph algorithms, allowing enumeration of valid edges in amortized $O(d^{2}\log\left(d\right))$ time while maintaining the clique/separator representation needed to compute local score changes throughout the search.

To represent the current structure and test the admissibility of local moves (i.e., preserving decomposability), we maintain a junction tree T=(K,F) associated with the current chordal graph *G*, where K is the set of maximal cliques of *G* and F is a set of edges connecting these cliques so that *T* satisfies the clique intersection property (i.e., *T* is a clique tree representation of *G*). We keep a single junction tree representation of the current graph and update it dynamically after each accepted move; all clique and separator terms (including their multiplicities) are computed from this maintained junction tree (equivalently, separator terms correspond to the multiset $\left\{K\cap K^{\prime }:\left(K,K^{\prime }\right)\in F\right\}$). For prospective insertion of a non-existing edge $\left\{v_{i},v_{j}\right\}$, the method considers the closest cliques $K_{k}$ and $K_{l}$ in the current join tree such that $v_{i}\in K_{k}$ and $v_{j}\in K_{l}$ and uses a weight function $w:F\to Z_{+}$ on the join tree edges defined by

$$w\left(K_{k},K_{l}\right)=\left|K_{k}\cap K_{l}\right|.$$

Insertion is valid precisely when the minimum-weight edge along the path between $K_{k}$ and $K_{l}$ has a weight equal to $w(K_{k},K_{l})$, ensuring that a clique tree for the updated graph exists [9,15]. For deletion of an existing edge $\left\{v_{i},v_{j}\right\}$, validity is characterized more simply; $\left\{v_{i},v_{j}\right\}$ can be removed while preserving decomposability if and only if the current graph has exactly one maximal clique containing both $v_{i}$ and $v_{j}$ [9,15]. To make these checks efficient, the join tree is maintained using a dynamic tree representation based on splay trees, supporting link and cut and shortest-path queries in amortized O(log(d)) time. Since a chordal graph on *d* variables has at most *d* cliques, the required join tree operations inherit this bound [9,15].

Maintaining chordality throughout the search ensures that every intermediate graph admits a valid clique/separator decomposition, and thus score evaluation never leaves the decomposable regime the theory assumes. Moreover, the dynamic junction tree machinery makes it possible to enumerate and test candidate moves without resorting to expensive global triangulation at each step. With the learning procedure fully specified, attention can now shift to empirical behavior, or how well the method recovers edges from finite samples and how its runtime scales under repeated local updates.

The learning problem is reduced to three modular components: a decomposable information score, a local-move mechanism that preserves chordality, and a nonparametric estimator for the clique/separator copula contributions. This modularity is essential; it separates statistical modeling choices (how to estimate ψ(A)) from graph-theoretic constraints (among which moves are admissible) and from optimization strategy (greedy updates with local deltas). The next section validates this pipeline empirically on controlled synthetic settings and on a real dataset.

Let *k* be the number of folds used in the predictive log-score estimator of Section 3.3, let ω+1 denote the maximum clique size over graphs visited by the search, and write $N_{\mathrm{iter}}$ and $N_{\mathrm{cand}}^{\left(q\right)}$ for the number of greedy iterations and the number of admissible candidate moves at iteration *q*, respectively. For a fixed subset *A* with |A|≤ω+1, the *k*-fold predictive log-score ${\hat{\psi }}_{\mathrm{KDE}}\left(A\right)$ in Equation (14) is obtained by repeatedly evaluating the probit-KDE in Equation (13) on held-out indices $t\in J_{r}$ using all training indices $s\in J_{-r}$. A direct implementation therefore requires $O(n^{2}\left|A|\right)\leq O\left(n^{2}\omega \right)$ kernel evaluations per ${\hat{\psi }}_{\mathrm{KDE}}\left(A\right)$. Proposition 2 shows that each candidate edge $\left\{v_{i},v_{j}\right\}$ in $N(G^{\left(q\right)})$ requires only the four subsets, and thus one local score difference $\Delta_{ij}\left(G^{\left(q\right)}\right)$ can be computed in $O(n^{2}\omega )$ time up to a constant factor. Since the dynamic tree machinery of Section 3.4 yields all admissible candidates $N(G^{\left(q\right)})$ and supports the corresponding junction tree updates in amortized $O(d^{2}\log d)$ and O(logd) time, the dominant cost of iteration *q* is $O\left(d^{2}\log d\right)\;+\;O\left(N_{\mathrm{cand}}^{\left(q\right)}\;n^{2}\omega \right)$, and a worst-case bound for the whole greedy run is $O\left(N_{\mathrm{iter}}\;d^{2}\log d\right)\;+\;O\left(n^{2}\omega \sum_{t=1}^{N_{\mathrm{iter}}}N_{\mathrm{cand}}^{\left(q\right)}\right)$, highlighting that the overall complexity is driven primarily by the number of evaluated candidates and the $O(n^{2}\omega )$ cost of each KDE-based local score.

## 4. Experimental Evaluation

This section empirically assesses both the ability of the proposed copula information score to recover decomposable dependence graphs from finite samples and the computational behavior of the resulting greedy chordal search [9,15]. The experiments are designed to isolate the effect of the copula information score under controlled ground-truth decomposable graphs while also evaluating end-to-end performance within a realistic greedy chordal search loop. In addition to synthetic studies with a known truth, a real dataset experiment demonstrates how the learned structures can be applied in a practical setting. All experiments are based on undirected decomposable (chordal) structures and compare learned graphs using standard edge recovery metrics. The experiments were conducted on an 8-core Intel Core i7 machine with 16 GB of RAM and the code in Python 3.10.

### 4.1. Synthetic Data Generation

Now, we define a controlled data-generating process in which the ground-truth dependence structure is known and explicitly decomposable. The construction proceeds in two stages; the first involves sampling a random graph and then applying a chordal completion to guarantee decomposability, and the second involves sampling observations from a Gaussian copula Markov random field whose latent conditional independences match that chordal structure, while the observed univariate marginals are imposed by applying the corresponding quantile functions. This set-up provides a clean benchmark for recovery, since any deviation in the learned edges can be attributed to finite-sample effects, estimation error in the score, or search sub-optimality.

The number of variables d∈N and an edge probability ρ∈(0,1) are fixed. We first draw an Erdos–Rényi graph $G_{0}∼G\left(d,\rho \right)$ and then obtain a chordal (decomposable) ground-truth graph $G=\tau (G_{0})$ via a triangulation (chordal completion) procedure [29]. We treat the resulting chordal graph G=(V,E) with $V=\{v_{1},\ldots ,v_{d}\}$ as the ground-truth conditional-independence structure.

Conditional on *G*, we draw IID latent weights

$$Y_{ij}∼\mathrm{Gamma}\left(\alpha =2,\beta =1\right),\;1\leq i<j\leq d,$$

where α denotes the shape parameter such that $E[Y_{ij}]=\alpha /\beta$. Define the symmetric weighted adjacency matrix $W\in R^{d\times d}$ by

$$W_{ij}=\chi_{E}\left(\left\{v_{i},v_{j}\right\}\right)\;Y_{ij},\;i<j,\;W_{ji}=W_{ij},\;W_{ii}=0.$$

Let $D=\mathrm{diag}(d_{1},\ldots ,d_{d})$ be the diagonal degree matrix with $d_{i}=\sum_{k=1}^{d}W_{ik}$, and let $I_{d}$ denote the d×d identity matrix. We construct the precision matrix $Q\in R^{d\times d}$ as a ridge-regularized graph Laplacian:

$$Q=\nu I_{d}+D-W,\;\nu >0.$$

This matrix is strictly diagonally dominant and hence positive definite, and its off-diagonal sparsity pattern matches the edge set *E*, so it can be used as the precision matrix of a copula Gaussian graphical model [30,31]. Let $Q^{-1}$ denote the latent covariance matrix. Since a Gaussian copula is parameterized by a correlation matrix, we normalize $Q^{-1}$ to a correlation matrix using

$$S=\mathrm{diag}{\left(Q^{-1}\right)}^{1/2},$$

so that $S^{-1}Q^{-1}S^{-1}$ has a diagonal unit. Note that diagonal rescaling preserves the conditional independence graph; the standardized latent precision is SQS, which has the same zero pattern as *Q* while keeping the identifiability of the marginal scales. We generate IID latent vectors via

$$Z^{\left(1\right)},\ldots ,Z^{\left(n\right)}∼\mathrm{Multivariate}\;\mathrm{Normal}\left(0,\;{\left(SQS\right)}^{-1}\right),$$

and form pseudo-observations $U_{j}^{\left(t\right)}=\Phi \left(Z_{j}^{\left(t\right)}\right)\in \left(0,1\right)$, where Φ is the standard normal CDF.

Finally, we obtain the observed data by applying the PPF of a chosen univariate distribution component-wise:

$$X_{j}^{\left(t\right)}=F_{j}^{-1}\;\left(U_{j}^{\left(t\right)}\right),\;j\in \left\{1,\ldots ,d\right\},\;t\in \left\{1,\ldots ,n\right\}.$$

In maintaining the same pseudo-observations $\left\{U_{j}^{\left(t\right)}\right\}$ across marginal regimes (and applying the same marginal distribution to all coordinates), we consider three choices with distinct supports: (1) Normal(0,1) on R, for which $F^{-1}=\Phi^{-1}$ and therefore $X_{j}^{\left(t\right)}=\Phi^{-1}\left(U_{j}^{\left(t\right)}\right)=Z_{j}^{\left(t\right)}$; (2) Exponential(1) on [0,∞), using the exponential PPF; and (3) Beta(1/2,1/2) on [0,1], using the beta PPF.

By generating data from a Gaussian copula graphical model whose latent precision respects the chordal sparsity pattern, the experiment provides a well-understood baseline in which missing edges correspond to conditional independences in the latent Gaussian layer, while the three marginal regimes probe learning performance under unbounded real-valued, strictly positive, and bounded supports. This design isolates marginal heterogeneity; across regimes, we keep the same latent dependence graph (Gaussian copula and conditional independences) and modify only the one-dimensional marginals via component-wise monotone transforms.

### 4.2. Learning Objectives and Protocol

The experimental protocol is designed so that structure learning and evaluation are clearly separated. A train–test split decouples optimization of the graph structure from the assessment of generalization, while repeated random seeds quantify variability due to both sampling noise and random ground-truth draws. Two scoring strategies are compared within the same greedy chordal search mechanism, ensuring that observed differences can be attributed primarily to the scoring function rather than to the optimizer.

Each synthetic run used (d,n,ρ)=(30,2000,0.1), and we repeated the experiment over 20 random seeds. For each run, to decouple structure learning from held-out evaluation, the full sample index set {1,…,n} was split into disjoint training (70%) and test (30%) sets, denoted by $J_{\mathrm{tr}}$ and $J_{\mathrm{te}}$ with sizes $n_{\mathrm{tr}}:=\left|J_{\mathrm{tr}}\right|$ and $n_{\mathrm{te}}:=\left|J_{\mathrm{te}}\right|$ (and $n=n_{\mathrm{tr}}+n_{\mathrm{te}}$), respectively. All cross-validation steps described in Section 3.3 were performed on the training set $J_{\mathrm{tr}}$ only. In the outer train and test evaluation, pseudo-observations for the test points were computed by applying the training empirical marginals to the test values (fit on train and transform test). Structure learning was performed via greedy forward and backward search over decomposable graphs, starting from the empty graph and applying only decomposability-preserving single-edge insertions and deletions until no improving move existed.

We compared three structure-learning procedures. NG-LL [9] and NG-CBIC both use the same greedy chordality-preserving search described above, differing only in the score used to accept or reject candidate edge insertions and deletions. In particular, NG-LL uses a predictive (cross-validated) log-likelihood score built from nonparametric KDE estimates of the clique and separator marginals under the decomposable factorization (i.e., it aggregates clique log-likelihood contributions and subtracts separator contributions). This provides a direct contrast with NG-CBIC, which scores candidate graphs using our copula information objective (copula entropy and mutual information terms) together with a CBIC-type complexity penalty. The third baseline, IC-ILP [10], operates on a discretized version of the data obtained by equal-frequency (quantile) binning of each variable and learns a decomposable structure by solving the resulting discrete optimization problem that maximizes the BIC score over decomposable graphs via an ILP-based procedure. This was evaluated on the same train and test splits and seeds.

Within the NG framework, we considered two alternative scoring functionals for greedy structure learning over decomposable graphs: a predictive-assessment criterion based on clique/separator KDEs evaluated using *k*-fold cross-validated log-likelihood under clique/separator KDEs (NG-LL) and a copula information criterion with a CBIC-type complexity penalty of the form in Equation (10) using λ(A)=|A|(|A|+1)/2.

This protocol enables the study of both accuracy and stability, namely accuracy through edge recovery metrics against a known truth and stability through variability across seeds under fixed (d,n,ρ) values. Because the search started from the empty graph and stopped at a local optimum, the results also indirectly reflect how informative and well-behaved the local deltas were under each score. The following subsection formalizes the recovery metrics used to quantify these outcomes.

### 4.3. Graph Recovery Metrics

The metrics used to compare the learned graph $\hat{G}$ with the ground-truth graph *G* are defined as follows. Because the output is an undirected structure, edge-wise discrepancies were summarized using both an edit distance SHD and classification-style measures (precision, recall, and F1 score) computed from TP, FP, and FN. Taken together, these metrics distinguish between conservative learners (high precision, low recall) and aggressive learners (high recall, low precision), a contrast that is relevant when scoring functions include explicit complexity penalties.

Let $W\in \{{0,1\}}^{d\times d}$ denote the symmetric adjacency matrix of an undirected graph G=(V,E), with $W_{ii}=0$ and $W_{ij}=W_{ji}=1\Leftrightarrow \left\{v_{i},v_{j}\right\}\in E$. Let *G* be the ground truth and $\hat{G}$ be the learned graph, with adjacency matrices *W* and $\hat{W}$.

We used the standard SHD, i.e., the number of edge additions and deletions needed to transform $\hat{G}$ into *G* [32]. For undirected graphs, this equals

(15) $$\mathrm{SHD}\left(G,\;\hat{G}\right)=\frac{1}{2}\sum_{i=1}^{d}\sum_{j=1}^{d}\left|W_{ij}-{\hat{W}}_{ij}\right|.$$

Define the (upper-triangular) edge sets *E* and $\hat{E}$ and the counts:

$$\mathrm{TP}=|\hat{E}\cap E\left|,\;\mathrm{FP}=\right|\hat{E}\setminus E\left|,\;\mathrm{FN}=\right|E\setminus \hat{E}|.$$

Then, we have

$$\mathrm{Precision}=\frac{\mathrm{TP}}{\mathrm{TP}+\mathrm{FP}},\;\mathrm{Recall}=\frac{\mathrm{TP}}{\mathrm{TP}+\mathrm{FN}},\;F1=\frac{2\;\mathrm{Precision}\cdot \mathrm{Recall}}{\mathrm{Precision}+\mathrm{Recall}}.$$

The combination of SHD and the F1 score provides complementary views; SHD measures absolute disagreement in edge sets, while the F1 score balances false positives and false negatives in a normalized way. Reporting multiple metrics is especially useful here because different scores may trade off sparsity and fit differently, even when optimized by the same greedy procedure. With these definitions in place, the evaluation extends beyond controlled simulations to a real dataset, where interpretability considerations become central.

### 4.4. Real Data Experiment

This subsection applies the proposed methodology to a gene expression dataset in which airway epithelial samples are stratified by smoking status (never, former, or current), using the study design and cohort described by Spira et al. [33]. The objective was to assess whether the learning pipeline produced a stable and interpretable chordal dependence summary in the presence of biological heterogeneity and measurement noise, rather than claim causal mechanisms or definitive regulatory structure. Accordingly, the workflow included standard diagnostic checks to characterize dominant variation and identify potential artifacts before fitting a decomposable model. To limit dimensionality (100 variables) and focus on the smoking-related signal, the analysis selected a fixed number of discriminative probes using an ANOVA-type screening criterion, after which structure learning was performed on the reduced probe set.

The learned chordal graph was interpreted as an exploratory conditional (in)dependence representation intended to support downstream biological hypothesis generation under a decomposability constraint. Because microarray platforms can map multiple probes to the same gene, probe-level edges are optionally aggregated to gene-level edges using platform annotation, which improves interpretability and reporting without altering the underlying score, estimator, or search procedure. In this cohort, smoking was reported to induce broad transcriptional changes in airway epithelial cells, including the upregulation of xenobiotic metabolism and redox-related genes, which motivates examining whether highly connected nodes in the learned dependence graph correspond to these functional categories. Overall, the real-data analysis complements the synthetic experiments by demonstrating the feasibility of chordal structure learning and by illustrating how annotation-aware post-processing can improve communication of graph structure in applied transcriptomic settings.

## 5. Results and Discussion

**Figure 1:**
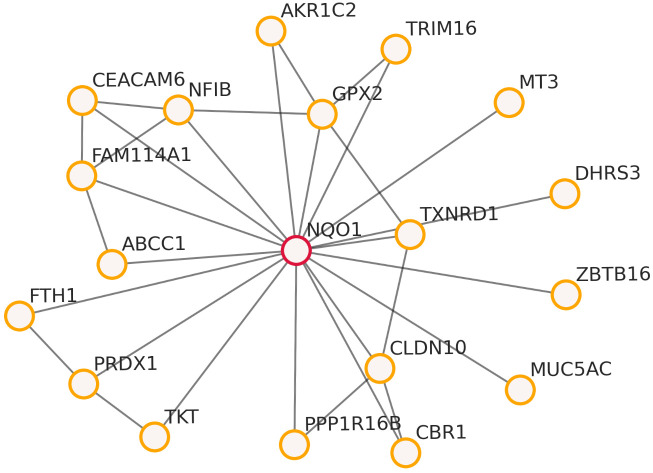
NQO1-centered ego network from the learned airway epithelial dependence graph. Radius-1 ego-graph centered at NQO1 extracted from the decomposable chordal graph learned on the airway epithelial microarray dataset after the real-data preprocessing and feature-screening protocol [33]. Nodes correspond to genes, and edges denote undirected conditional (in)dependence relationships in the fitted model (interpretable as co-expression associations after conditioning on the rest of the selected variables), highlighting a redox and detoxification neighborhood (e.g., GPX2, TXNRD1, PRDX1, and ABCC1) that is statistically linked to epithelial markers such as CEACAM6 and CLDN10.

This section evaluates the proposed copula information learning criterion in both controlled and applied settings, emphasizing empirical structure recovery and qualitative interpretability of the learned decomposable graphs. We first report a synthetic benchmark (Table 1), where the ground-truth chordal structure is known and standard graph-recovery metrics (e.g., F1 score and SHD) can be computed directly. In this benchmark, we additionally vary the univariate marginals (Gaussian, exponential, and beta) via component-wise monotone transforms while holding the copula (and thus the ground-truth conditional independence structure) fixed, directly reflecting the paper’s motivation regarding marginal heterogeneity. We then present a real gene expression application based on airway epithelial samples stratified by smoking status [33], using the learned network structure as an exploratory conditional (in)dependence summary and illustrating key local patterns via a hub-centered sub-network visualization (Figure 1).

### 5.1. Synthetic Data

Table 1 reports the structure recovery performance on a chordal synthetic benchmark with d=30 variables and n=2000 observations, averaged over 20 random seeds. The three marginal blocks (Gaussian, exponential, and beta) were generated by reusing the same pseudo-observations and applying only component-wise monotone quantile transforms, so the copula (and hence the ground-truth conditional independence structure) was held fixed across marginal families. This design separates two effects: (1) Monte Carlo variability of a learning criterion when the dependence structure is unchanged. In particular, methods that depend only on ranks or pseudo-observations recover the same graph within each seed across marginal families, whereas methods that score densities on the original scale can change with the marginal regime.

This pattern is visually clear in Table 1. NG-CBIC was essentially unchanged across Gaussian, exponential, and beta marginals (e.g., identical F1 scores and SHD across families), directly illustrating margin invariance, while NG-LL varied across the same marginal blocks, reflecting sensitivity to marginal distributions. Across all metrics, the proposed NG-CBIC achieved the strongest overall recovery, with the highest F1 score (0.86±0.04) and the lowest SHD (22.65±10.86), and its metrics were identical across the three marginal families under this protocol. In contrast, NG-LL varied substantially with the marginal regime; it attained an F1=0.76±0.05 with SHD=45.35±9.42 under Gaussian marginals but dropped to F1=0.66±0.06 with SHD=51.25±9.02 under exponential marginals. Finally, IC-ILP attained the lowest F1 score (0.47±0.08) and the highest SHD (62.30±20.31), despite its high precision (0.89±0.06), indicating a conservative regime dominated by false negatives.

The precision and recall profiles clarify these differences. NG-LL exhibited high recall under normal marginals (0.88±0.06) but relatively low precision (0.68±0.08), consistent with an edge-including behavior that favors sensitivity at the cost of false discoveries. The proposed NG-CBIC reversed this pattern by achieving extremely high precision (0.94±0.03) while maintaining strong recall (0.80±0.07), which directly explains its superior F1 score and SHD. IC-ILP was the most conservative method (recall 0.32±0.08), and this under-selection dominated its overall error rate, even though its precision remained high.

In Table 1, both IC-ILP and NG-CBIC exhibited identical performance across the normal, exponential, and beta marginal regimes. For IC-ILP, this was expected because equal-frequency (quantile) binning is determined by the ordering of the sample (empirical ranks) [34]. Since ranks are invariant under strictly monotone marginal transformations [35], the discretized dataset, and therefore the ILP objective and recovered graph, remained unchanged across marginal families generated by component-wise strictly increasing transforms of the same latent pseudo-observations. NG-CBIC was computed from pseudo-observations (empirical copula transforms) and thus depended only on ranks. Under the same protocol, this yielded the same learned graph across marginal families for each seed for both methods. Accordingly, the reported standard deviations summarize the Monte Carlo variability across random seeds (independent datasets), rather than variation across marginal families.

The runtime results show that IC-ILP was fastest, while NG-CBIC achieved higher accuracy than NG-LL at a similar runtime. This is consistent with the score and penalty combination leading to fewer unproductive edge modifications during the same chordality-preserving greedy search, thereby improving recovery without increasing the overall computational burden.

Dependence-focused scoring improved structural fidelity. The synthetic results demonstrate that optimizing decomposable structures using copula information with a CBIC-type penalty yielded substantially fewer structural errors than a likelihood-driven nonparametric baseline. In particular, NG-CBIC approximately halved the SHD relative to NG-LL while simultaneously increasing the F1 score. This improvement is consistent with the design goal of isolating dependence from one-dimensional marginal effects, which is central to copula-based information measures. The key implication is that for continuous-variable structure learning, a dependence-only objective can yield more reliable edge sets than directly optimizing the predictive likelihood of multivariate KDE factors in cliques and separators.

The copula formulation provides invariance to marginal artifacts. A central theoretical advantage of the proposed approach is that mutual information can be expressed as (negative) copula entropy so that the leading fit term depends only on the copula density rather than on marginal densities. This property makes the score invariant to strictly monotone transformations of individual variables (i.e., reparameterizations that preserve ranks), which directly targets conditional dependence rather than the marginal scale and shape. In practical terms, this invariance mitigates a common failure mode in continuous modeling where marginal irregularities can be spuriously absorbed by additional edges when the likelihood is optimized under flexible, nonparametric density models. The empirical signature of this effect appears in Table 1; NG-LL increased recall but paid a large precision cost, while NG-CBIC maintained high recall without inducing analogous false positive inflation.

Regularization explains the precision gains and stabilizes greedy search. The large precision jump from NG-LL to the proposed method indicates that explicit complexity control is essential, even when the search space is restricted to decomposable (chordal) graphs. Because decomposable models admit clique/separator factorizations, penalties that decompose over cliques and separators preserve locality, enabling score differences to be computed from small set changes induced by chordality-preserving edge edits. This locality is not merely computationally convenient; it also shapes the optimization landscape by discouraging diffuse sequences of small likelihood gains that accumulate into overly dense graphs. The resulting behavior is empirically favorable here; the proposed method sacrifices some recall relative to NG-LL but achieves substantially lower SHD results, meaning that the removed edges are predominantly false positives rather than true dependencies.

Nonparametric copula estimation requires explicit treatment of boundary bias. The statistical reliability of copula entropy estimation hinges on estimating densities supported on multidimensional unit hypercubes, where naive kernel smoothing suffers from boundary distortion. The probit transformation strategy addresses this issue by mapping pseudo-observations to unlimited supports, applying KDE in an unconstrained domain, and correcting via the Jacobian upon mapping back, which is a standard approach in nonparametric copula density estimation. This choice is consistent with the need for repeated evaluation of many overlapping clique/separator subsets during greedy search, where even mild systematic bias can accumulate into persistent edge selection errors. In this sense, the proposed estimator is not an auxiliary detail but a prerequisite for the observed precision improvements, because it reduces the tendency of the score to overreact to artifacts near the boundaries of the copula domain.

Decomposability constrains expressiveness but enables scalable and well-defined learning. Restricting to decomposable graphs is a structural bias that trades representational flexibility for algorithmic tractability and score locality. On the positive side, chordality guarantees a junction tree representation, making global quantities decomposable and enabling efficient updates under local edits, which is exactly the regime required by stepwise search procedures. On the negative side, non-chordal dependence patterns must be approximated by a chordal completion, and thus learned edges can reflect the best chordal surrogate rather than the exact original sparse structure when the data-generating mechanism is not decomposable. The present synthetic protocol explicitly constructs chordal ground truths via triangulation, and thus the reported gains primarily reflect differences in scoring and estimation rather than a mismatch between the truth and the hypothesis class.

Discrete or coarsening-based approaches can underperform in continuous problems. The conservative behavior of IC-ILP (precision of 0.89 with recall of 0.32) suggests that its optimization biases favor sparse backbones and may fail to recover weaker conditional dependencies in continuous settings when the scoring machinery is not tailored to copula- or density-based continuous criteria. More broadly, discrete decomposable model learning pipelines are susceptible to quantization effects when continuous variables are binned, because discretization can discard dependence information and introduce bin edge artifacts that propagate into structure learning. This issue is especially relevant in scientific measurements, where binning choices can dominate the effective sample size within contingency tables (e.g., biomedical signals). Consequently, the results support a principled division of labor: coarsening and ILP methods remain valuable for large-scale discrete decomposable learning, whereas copula information scoring provides a more appropriate inductive bias for continuous dependence learning.

### 5.2. Real Data

Inspecting the immediate neighborhood of NQO1 in Figure 1 is useful because, in a learned conditional (in)dependence graph, a hub’s one-hop ego-network provides a compact view of the strongest local association structure around a biologically salient marker without requiring global, hard-to-parse topology. In the implied radius-one ego-network figure, NQO1 is the highest-degree node, connecting to an antioxidant- and redox-associated set (GPX2, TXNRD1, and PRDX1), a small metabolic motif (TKT and FTH1 linked via PRDX1 and also adjacent to NQO1), and an epithelial marker- and phenotype-associated branch (CEACAM6, CLDN10, and MUC5AC). Two aspects are especially worth highlighting within this local view; TRIM16 connects into the antioxidant pocket via TRIM16–GPX2, and MT3 (a metallothionein) appears directly adjacent to NQO1, which is notable given that metallothionein behavior can differ between acute and chronic smoke exposure contexts.

In the manuscript’s real-data experiment, the airway epithelial gene expression data serve as an applied demonstration for the proposed decomposable structure-learning framework, so the edges should be read strictly as conditional (in)dependence–co-expression relationships under a tractable chordal model rather than as regulatory directionality. At a coarse functional level, the NQO1 neighborhood is consistent with smoking-associated airway epithelial transcriptional shifts reported in the classic bronchial brushing study by Spira et al. [33]. The same paper also reported increased epithelial adhesion markers in smokers, which aligns descriptively with CEACAM6 and CLDN10 sitting in the same immediate neighborhood as the redox/xenobiotic module.

Beyond this corroborative signal, the TRIM16–GPX2 linkage is plausibly interesting because TRIM16 has been described as participating in oxidative and proteotoxic stress programs through the p62–KEAP1–NRF2 axis, making its proximity to a canonical detox or antioxidant pocket a coherent, non-mechanistic highlight [36]. Likewise, MT3’s appearance near NQO1 can be highlighted cautiously because airway metallothionein genes have been reported to show discordant responses across exposure regimes, and thus a local association between MT3 and an oxidative stress hub could be context-sensitive rather than universally expected [37]. From a statistical standpoint, these compact neighborhood-level findings illustrate what the proposed method can improve in network analysis (such as in gene expression analysis): dependence-focused edge selection that is more robust to marginal artifacts (via copula-based information measures) while remaining interpretable and computationally manageable through decomposable structure constraints.

### 5.3. Limitations

This work deliberately focused on DM because chordality is the structural condition that enables a junction tree representation and avoids computation of the intractable partition function [3,4]. That representation is what makes clique/separator factorization available and supports tractable exact inference together with genuinely local score updates during structure search, which are central to the approach developed here. As a consequence, the method is best understood as learning an interpretable tractable of dependence rather than an unconstrained representation of a conditional-independence structure that may exist in the data [4].

The chordal restriction implies an unavoidable modeling bias when the data-generating dependence is not itself chordal. In real applications, non-chordal dependence patterns may be common, and a decomposable model can only approximate such patterns within the chordal family. In practice, this approximation often manifests through additional edges needed to maintain chordality, which can reduce sparsity and complicate interpretation because some edges may serve primarily to support a chordal scaffold rather than indicate a direct scientific relationship.

The restriction to decomposable graphs also interacts with finite-sample estimation through the clique/separator organization of the score. Section 3 instantiates clique/separator copula entropy terms using pseudo-observations, a probit-based transformation, and predictive evaluation to mitigate boundary effects on the unit hypercube. Even with these design choices, the reliability of repeated nonparametric copula entropy evaluations can deteriorate as clique and separator sizes grow, because the estimation problems become higher-dimensional, more bandwidth-sensitive, and more variable in finite samples. When chordal approximations induce larger cliques than one would obtain under an unconstrained representation, this dimensionality effect can translate into increased runtimes and additional variability in local score comparisons during a search.

The optimization procedure reflects a deliberate trade-off between scalability and global optimality. The proposed method uses greedy, chordality-preserving single-edge moves to keep the search computationally feasible while maintaining decomposability at every step. As with greedy procedures in combinatorial graph spaces, this choice does not guarantee convergence to a global optimum of the objective over the decomposable class, and finite-sample numerical behavior can depend mildly on the maintained junction tree representation because separator contributions enter according to that representation’s induced multiplicities, even though the population-level decomposition is a property of the underlying decomposable graph.

Finally, copula-based modeling provides a powerful tool for constructing joint distributions. However, researchers employing this technique should be aware of several limitations. One challenge arises when using copulas with discrete marginal distributions. Sklar’s theorem [11] demonstrates that in such cases, a unique copula representation for the joint distribution is not guaranteed. This lack of uniqueness can translate into identifiability issues [38] for the model parameters.

## 6. Conclusions

This work studied structure learning for continuous decomposable (chordal) Markov random fields using an information-theoretic objective defined on copulas to learn dependence graphs that are robust to marginal artifacts. The central methodological conclusion is that combining copula-based mutual information principles with clique/separator decomposability yields a scoring rule that remains compatible with chordality-preserving local search while targeting dependence rather than a marginal shape. This conclusion directly addresses the motivating gap that likelihood-driven nonparametric decomposable models can be accurate yet sensitive to marginal irregularities and smoothing choices in high-variance continuous data.

The empirical findings support this dependence-focused perspective on a controlled chordal synthetic benchmark. On this benchmark, the proposed NG-CBIC method achieved the highest F1 score and the lowest SHD, indicating substantially improved recovery of the ground-truth edge set. Compared with the predictive assessment likelihood baseline NG-LL, NG-CBIC approximately halved the SHD while improving the F1 score, which is consistent with reduced false positive inflation under marginally robust scoring. Compared with IC-ILP, NG-CBIC improved the recall while maintaining high precision, showing that the information-based criterion is less prone to the under-selection behavior observed in sparse-backbone learners.

One practical implication is that for continuous-variable structure learning under heterogeneous marginals, copula information scoring with an explicit complexity penalty can yield graphs that are both more accurate and more interpretable than graphs learned by optimizing the nonparametric likelihood alone. A second practical implication is that stable copula entropy estimation is not an implementation detail but a necessary component for reliable edge decisions in repeated local updates, motivating the use of probit-based boundary correction for KDE on multidimensional unit hypercubes. The real-data experiment further indicates that the approach can be applied end to end to noisy gene expression measurements to produce an exploratory dependence summary that supports domain interpretation while remaining explicit about its non-causal status.

Several directions appear particularly important for future works, such as strengthening the complexity control to more directly discourage large cliques while preserving clique/separator additivity; adding stability assessments (for instance, via resampling) to identify edges that are not robust under finite-sample variability; complementing greedy search with junction tree-based sampling to quantify uncertainty over decomposable structures; and developing faster or more stable estimators tailored to repeated clique/separator evaluation. Additionally, a theory is needed to characterize when chordal restrictions and chordal completions affect edge recovery under copula-based scores, especially beyond treewidth-one regimes, where sharp recoverability results are currently better understood. Uncertainty quantification over decomposable structures could be integrated by combining the present dependence score with modern junction tree samplers for decomposable graphs, enabling posterior summaries or stability measures rather than a single selected graph. Scaling the copula entropy evaluation to larger cliques and higher dimensions remains an open computational problem. In addition to parallel programming, the clique/separator decomposition suggests a natural fit with distributed and federated settings, where copula entropy contributions can be computed locally and combined without sharing raw observations, motivating new approximations and caching strategies that preserve the locality exploited by chordal search. Finally, extending the framework to non-IID regimes (e.g., temporal dependence) would broaden applicability but would also require scores and estimators that remain valid under dependence while preserving decomposable updates.

## Figures and Tables

**Table 1:** Synthetic benchmark across marginal families from three distinct supports (Section 4.1): edge recovery and runtime for decomposable structure learning. Across the three marginal families, we reused the same pseudo-observations and applied only component-wise quantile transforms, so the copula (and ground-truth conditional independence structure) was held fixed. Mean ± standard deviation over 20 random seeds for a chordal synthetic experiment with d=30 variables and n=2000 samples (with the specified edge probability setting and train and test protocol described in Section 4.2, comparing NG-LL [9] (nonparametric log-likelihood), NG-CBIC (proposed copula information score with CBIC-type penalty), and IC-ILP [10] (discrete or coarsening ILP baseline with quantile binning). Metrics include F1 score, precision, recall, SHD, and wall clock time in seconds. NG-CBIC rows are identical across marginal families, visually demonstrating margin invariance. NG-LL varies across marginals, indicating marginal sensitivity.

Method	Marginals	F1 Score	Precision	Recall	SHD	Time [s]
NG–LL	Gaussian	0.76±0.05	0.68±0.08	0.88±0.06	45.35±9.42	33.67±7.15
NG–CBIC	Gaussian	0.86±0.04	0.94±0.03	0.80±0.07	22.65±10.86	22.25±4.63
IC–ILP	Gaussian	0.47±0.08	0.89±0.06	0.32±0.08	62.3±20.31	8.73±1.51
NG–LL	Exponential	0.66±0.06	0.77±0.07	0.59±0.09	51.25±9.02	16.16±1.55
NG–CBIC	Exponential	0.86±0.04	0.94±0.03	0.80±0.07	22.65±10.86	20.19±3.85
IC-ILP	Exponential	0.47±0.08	0.89±0.06	0.32±0.08	62.3±20.31	8.69±1.40
NG–LL	Beta	0.73±0.03	0.68±0.05	0.81±0.08	47.70±13.15	26.60±4.92
NG–CBIC	Beta	0.86±0.04	0.94±0.03	0.80±0.07	22.65±10.86	21.05±3.82
IC-ILP	Beta	0.47±0.08	0.89±0.06	0.32±0.08	62.3±20.31	7.77±1.37

## Data Availability

The data presented in this study are openly available in Gene Expression Omnibus Database at https://doi.org/10.1007/978-1-4939-3578-9_5.

## Abbreviations

The following abbreviations are used in this manuscript:

ANOVA	Analysis of variance
BIC	Bayesian information criterion
BN	Bayesian network
CBIC	Continuous Bayesian information criterion
CDF	Cumulative distribution function
CV	Cross-validation
DAG	Directed acyclic graph
DM	Decomposable model
IC	Iterative coarsening
IID	Independent and identically distributed
ILP	Integer linear programming
KDE	Kernel density estimator
LL	Log-likelihood
MRF	Markov random field
NG	Non-parametric greedy
PDF	Probability density function
PGM	Probabilistic graphical model
PPF	Percent-point function
SHD	Structural Hamming distance
UG	Undirected graph
**Symbols**	
R	Real numbers
$R_{+}$	Nonnegative reals
N	Natural numbers
$Z_{+}$	Nonnegative integers
E	Expectation
*d*	Dimension (number of variables or nodes)
*n*	Sample size
$J_{\mathrm{tr}}$	Training index set
$J_{\mathrm{te}}$	Test index set
$n_{\mathrm{tr}}$	Training sample size
$n_{\mathrm{te}}$	Test sample size
*k*	Fold count in *k*-fold cross-validation
i,j	Variable indices i,j∈{1,…,d}
*t*	Sample index t∈{1,…,n}
(Ω,A,P)	Probability space
$X={\left(X_{1},\ldots ,X_{d}\right)}^{T}$	Continuous random vector in $R^{d}$
$F_{X},\;F\left(x\right)$	Joint CDF of X
$p_{X}$	Joint PDF of X
$F_{i}$	Marginal CDF of $X_{i}$
$p_{i}$	Marginal PDF of $X_{i}$
*C*	Copula function
*c*	Copula density
$U_{i}$	Distributional transform $U_{i}=F_{i}\left(X_{i}\right)$
U	Transformed random vector $U={\left(U_{1},\ldots ,U_{d}\right)}^{T}$
u	Generic point in ${\left[0,1\right]}^{d}$
*h*	Differential entropy
$h_{c}$	Copula entropy
*I*	Mutual information
*A*	Index set A⊆{1,…,d}
|·|	Cardinality of a set, absolute value
$A^{c}$	Complement of *A* in {1,…,d}
$\pi_{A}$	Coordinate projection $\pi_{A}:R^{d}\to R^{\left|A\right|}$
$X_{A}$	Subvector $X_{A}=\pi_{A}\left(X\right)$
$X_{-A}$	Complement subvector $X_{-A}=X_{A^{c}}$
$U_{A}$	Subvector $U_{A}=\pi_{A}\left(U\right)$
*G*	Undirected graph
*V*	Vertex set $V=\left\{v_{1},\ldots ,v_{d}\right\}$
*E*	Edge set E⊆V×V
$v_{i}$	Vertex
K	Maximal clique set K
$K_{l}$	*l*-th maximal clique in in running-intersection ordering
$S_{l}$	*l*-th separator set in running-intersection ordering ($S_{1}=\emptyset$)
*m*	Number of maximal cliques
η	Predecessor index in running-intersection ordering
γ	Copula marginal index set γ (for γ marginal)
$\alpha_{l}$	Bijection $\alpha_{l}:\left\{1,\ldots ,|K_{l}|\right\}\to K_{l}$ (argument order)
$\gamma_{l}$	Separator coordinate positions $\gamma_{l}=\alpha_{l}^{-1}\left(S_{l}\right)$
$c_{A}$	Copula density on margin set indexed by *A*
$c_{K_{l}}$	Clique copula density
$c_{S_{l}}$	Separator copula density $c_{S_{l}}\equiv c_{K_{l}}^{\gamma_{l}}$
ψ	Local information functional
I	Global information
Λ	Complexity functional
λ	Local complexity term
$S_{\mathrm{CBIC}}$	CBIC-type score
$\Delta_{ij}$	Local move score change
κ	Local score functional
χ	Indicator function
$\hat{F}$	Empirical marginal CDF
$\hat{U}$	Pseudo-observation
Φ	Standard normal CDF
ϕ	Standard normal PDF
$\hat{Z}$	Probit-transformed pseudo-observation
θ	Global bandwidth vector $\theta \in R_{+}^{d}$
$\theta_{A}$	Bandwidth subvector
*g*	Gaussian kernel
$\hat{c}$	Copula density estimator
$J_{-r}$	*k*-fold training index set
$\hat{\psi }$	Local information estimator
$G_{\mathrm{dec}}$	Decomposable graph class
N(G)	Valid neighborhood $N\left(G\right)\subset G_{\mathrm{dec}}$
*T*	Junction tree T=(K,F)
F	Clique tree edge set
*w*	Join tree edge weight
ρ	Erdos–Rényi edge probability
τ	Triangulation/chordal completion map
*D*	Degree matrix
$I_{d}$	Identity matrix of size *d*
*Q*	Precision matrix
ν	Ridge parameter
*W*	Adjacency matrix
$\hat{W}$	Estimated adjacency matrix
$\mathrm{SHD}(G,\hat{G})$	Structural Hamming distance between *G* and $\hat{G}$

## References

[1] Rios F.L., Moffa G., Kuipers J. (2025). Benchpress: A Versatile Platform for Structure Learning in Causal and Probabilistic Graphical Models. J. Stat. Softw..

[2] Kitson N.K., Constantinou A.C., Guo Z., Liu Y., Chobtham K. (2023). A survey of Bayesian Network structure learning. Artif. Intell. Rev..

[3] Brémaud P. (2020). Markov Chains. Gibbs Fields, Monte Carlo Simulation and Queues.

[4] Pearl J., Pearl J. (1988). Chapter 3—MARKOV AND BAYESIAN NETWORKS: Two Graphical Representations of Probabilistic Knowledge. Probabilistic Reasoning in Intelligent Systems.

[5] Huang Y., Xiang Y. (1999). Learning Bayesian networks by learning decomposable Markov networks first. Engineering Solutions for the Next Millennium. 1999 IEEE Canadian Conference on Electrical and Computer Engineering (Cat. No.99TH8411).

[6] Olsson J., Pavlenko T., Rios F.L. (2022). Sequential sampling of junction trees for decomposable graphs. Stat. Comput..

[7] Katiyar A., Basu S., Shah V., Caramanis C., Camps-Valls G., Ruiz F.J.R., Valera I. (2022). Recoverability Landscape of Tree Structured Markov Random Fields under Symmetric Noise. Proceedings of the 25th International Conference on Artificial Intelligence and Statistics, Virtual, 28–30 March 2022.

[8] Pfeifer D., Kovács E.A. (2024). Vine copula structure representations using graphs and matrices. Inf. Sci..

[9] Schwaighofer A., Dejori M., Tresp V., Stetter M., de Sá J.M., Alexandre L.A., Duch W., Mandic D. (2007). Structure Learning with Nonparametric Decomposable Models. Proceedings of the Artificial Neural Networks—ICANN 2007.

[10] Orfanides G., Pérez A., Jaeger M., Nielsen T.D. (2020). Learning decomposable models by coarsening. Proceedings of the 10th International Conference on Probabilistic Graphical Models, Skørping, Denmark, 23–25 September 2020.

[11] Sklar M. (1959). Fonctions de Répartition à n Dimensions et Leurs Marges. Ann. l’ISUP.

[12] Ma J., Sun Z. (2011). Mutual Information Is Copula Entropy. Tsinghua Sci. Technol..

[13] Lasserre M., Lebrun R., Wuillemin P.H. (2021). Learning Continuous High-Dimensional Models using Mutual Information and Copula Bayesian Networks. Proc. AAAI Conf. Artif. Intell..

[14] Geenens G., Charpentier A., Paindaveine D. (2017). Probit transformation for nonparametric kernel estimation of the copula density. Bernoulli.

[15] Ibarra L. (2008). Fully dynamic algorithms for chordal graphs and split graphs. ACM Trans. Algorithms.

[16] Provost S.B., Zang Y. (2024). Nonparametric Copula Density Estimation Methodologies. Mathematics.

[17] Durante F., Sempi C. (2015). Principles of Copula Theory.

[18] Rønn-Nielsen A., Hansen E. (2014). Conditioning and Markov Properties.

[19] Desuó Neto L. (2025). Untying the Gordian Knot of Bayesian Inference in Markov Networks. Ph.D. Thesis.

[20] Kraskov A., Stögbauer H., Grassberger P. (2004). Estimating mutual information. Phys. Rev. E.

[21] Zhu C., Byrd R.H., Lu P., Nocedal J. (1997). Algorithm 778: L-BFGS-B: Fortran subroutines for large-scale bound-constrained optimization. ACM Trans. Math. Softw..

[22] Byrd R.H., Lu P., Nocedal J., Zhu C. (1995). A Limited Memory Algorithm for Bound Constrained Optimization. SIAM J. Sci. Comput..

[23] Morales J.L., Nocedal J. (2011). Remark on “algorithm 778: L-BFGS-B: Fortran subroutines for large-scale bound constrained optimization”. ACM Trans. Math. Softw..

[24] Segers J. (2012). Asymptotics of empirical copula processes under non-restrictive smoothness assumptions. Bernoulli.

[25] Joe H. (1989). Estimation of entropy and other functionals of a multivariate density. Ann. Inst. Stat. Math..

[26] Hall P., Presnell B. (1999). Density Estimation under Constraints. J. Comput. Graph. Stat..

[27] Stone M. (1977). An Asymptotic Equivalence of Choice of Model by Cross-Validation and Akaike’s Criterion. J. R. Stat. Soc. Ser. B (Methodol.).

[28] Arlot S., Lerasle M. (2016). Choice of V for V-Fold Cross-Validation in Least-Squares Density Estimation. J. Mach. Learn. Res..

[29] Berry A., Blair J.R.S., Heggernes P., Peyton B.W. (2004). Maximum Cardinality Search for Computing Minimal Triangulations of Graphs. Algorithmica.

[30] Dempster A.P. (1972). Covariance Selection. Biometrics.

[31] Jones B., West M. (2005). Covariance decomposition in undirected Gaussian graphical models. Biometrika.

[32] Tsamardinos I., Brown L.E., Aliferis C.F. (2006). The max-min hill-climbing Bayesian network structure learning algorithm. Mach. Learn..

[33] Spira A., Beane J., Shah V., Liu G., Schembri F., Yang X., Palma J., Brody J.S. (2004). Effects of cigarette smoke on the human airway epithelial cell transcriptome. Proc. Natl. Acad. Sci. USA.

[34] ouriarhli B., Benyacoub B., Benazza H. (2026). An Evaluation of Discretization Techniques for HMM-Based Classifiers. Stat. Optim. Inf. Comput..

[35] Brunner E., Puri M.L. (1996). 19 Nonparametric methods in design and analysis of experiments. Design and Analysis of Experiments.

[36] Jena K.K., Kolapalli S.P., Mehto S., Nath P., Das B., Sahoo P.K., Ahad A., Syed G.H., Raghav S.K., Senapati S. (2018). TRIM16 controls assembly and degradation of protein aggregates by modulating the p62-NRF2 axis and autophagy. EMBO J..

[37] Billatos E., Faiz A., Gesthalter Y., LeClerc A., Alekseyev Y.O., Xiao X., Liu G., ten Hacken N.H.T., Heijink I.H., Timens W. (2018). Impact of acute exposure to cigarette smoke on airway gene expression. Physiol. Genom..

[38] Genest C., Nešlehová J. (2007). A Primer on Copulas for Count Data. ASTIN Bull..

